# XenDB: Full length cDNA prediction and cross species mapping in *Xenopus laevis*

**DOI:** 10.1186/1471-2164-6-123

**Published:** 2005-09-14

**Authors:** Alexander Sczyrba, Michael Beckstette, Ali H Brivanlou, Robert Giegerich, Curtis R Altmann

**Affiliations:** 1 FSU College of Medicine, Department of Biomedical Sciences, 1269 W. Call Street, Tallahassee, FL 32306, USA; 2 AG Praktische Informatik, Technische Fakultät, Universität Bielefeld, D-33594 Bielefeld, Germany; 3 The Rockefeller University, Laboratory of Molecular Vertebrate Embryology, 1230 York Avenue, New York, NY 10021, USA

## Abstract

**Background:**

Research using the model system *Xenopus laevis *has provided critical insights into the mechanisms of early vertebrate development and cell biology. Large scale sequencing efforts have provided an increasingly important resource for researchers. To provide full advantage of the available sequence, we have analyzed 350,468 *Xenopus laevis *Expressed Sequence Tags (ESTs) both to identify full length protein encoding sequences and to develop a unique database system to support comparative approaches between *X. laevis *and other model systems.

**Description:**

Using a suffix array based clustering approach, we have identified 25,971 clusters and 40,877 singleton sequences. Generation of a consensus sequence for each cluster resulted in 31,353 tentative contig and 4,801 singleton sequences. Using both BLASTX and FASTY comparison to five model organisms and the NR protein database, more than 15,000 sequences are predicted to encode full length proteins and these have been matched to publicly available IMAGE clones when available. Each sequence has been compared to the KOG database and ~67% of the sequences have been assigned a putative functional category. Based on sequence homology to mouse and human, putative GO annotations have been determined.

**Conclusion:**

The results of the analysis have been stored in a publicly available database XenDB . A unique capability of the database is the ability to batch upload cross species queries to identify potential *Xenopus *homologues and their associated full length clones. Examples are provided including mapping of microarray results and application of 'in silico' analysis. The ability to quickly translate the results of various species into '*Xenopus*-centric' information should greatly enhance comparative embryological approaches.

Supplementary material can be found at .

## Background

Following the publication of the first automated cDNA sequencing study in 1991 demonstrating the utility of large scale random clone cDNA sequencing approaches [1], there has been a rapid and accelerating growth of such Expressed Sequence Tags (EST). The initial study of 600 partial human sequences has grown to more than 20.0 × 10^6 ^while more than 30 organisms have more than 100,000 sequences. To make sense of the resulting sequence, a variety of bioinformatic approaches have been developed to identify protein coding sequences and domains [2-4] and generate 'unigene' sets based on agglomerative clustering methods [5,6]. Clustering EST sequences is a widely used method for analyzing the transcriptome of a genome. Especially for organisms whose genome is not (yet) sequenced, the EST data is a valuable source of information. While enormously useful, most current analysis tools result in the loss of significant biological information such as alternatively spliced transcripts and polymorphisms [7-18]. Alternative splicing in particular plays important roles during both development and in the mature organism [7-15]. Moreover, most EST based approaches appear to overestimate the number of unique sequences compared to gene predictions based on whole genome sequencing efforts [19-22].

There are different approaches for EST clustering; the most commonly used being (1) each cluster represents a distinct gene, alternative transcripts of the same gene are grouped together into the same cluster. UniGene is one approach that uses this gene-based strategy [23-27]. (2) Alternative transcripts are represented by distinct clusters. Using genome assembly tools like CAP3 [28] or Phrap [29,30] results in such a clustering, as these tools cannot (and are not designed to) handle the kinds of differences in the EST sequences. (3) STACK [6] groups ESTs based on their tissue source first, and clusters are then generated for each tissue separately. Our approach first generates gene-oriented clusters and then attempts to generate separate contigs which potentially correspond to alternative transcripts.

The underlying principle for each of these approaches is a pairwise comparison of all sequences to identify common subsequences of a given length and identity that is subsequently used to group sequences into clusters. The types of pairwise comparisons result in a runtime that is quadratic in the number of sequences to be compared. To achieve better running times, most tools try to identify promising pairs of sequences by applying word-based algorithms, which consider the frequency of common words in each pair of sequences [31]. In any case these approaches have to compare all possible pairs of sequences, resulting in a running time that grows quadratically with the number of sequences. We have implemented a pipeline for rapid processing and clustering of EST data, based on enhanced suffix arrays [32-34]. Compared to other methods it reduces the running time tremendously. While we focus on generating gene-based clusters, we also assembled each cluster separately using CAP3 to generate consensus sequences for further analyses. Liang et al. evaluated Phrap, CAP3, TA-EST and TIGR Assembler and found in their analysis that CAP3 consistently out-performed the other programs [35]. We therefore chose CAP3 for cluster assembly.

All sequence and clustering information obtained with our approach was stored in a relational database system. To allow for extensive queries, GenBank annotations were incorporated including the library source, tissue type, cell type and developmental stage. Results of all sequence analyses performed on the consensus sequences were stored in the database. This way, comparative queries could be answered to identify e.g. full length clones, sequences unique to *X. laevis*, or shared between *Xenopus *and another organism. The comparative query also allows the identification of the set of *Xenopus *sequences most related to a set from another organism. Thus, the XenDB database is designed to address a critical issue facing many researchers: the comparison of genomic studies in one organism and their application to studies in another model organism. This task is faced by many laboratories attempting to extract the information gained in human, mouse, fly and worm microarray and library sequencing studies which often consist of large tables of genes.

While other databases such as UniGene [36] or TIGR Gene Indices [37] also provide collections of clustered ESTs, the unique batch functionality of mapping results from other organisms to *Xenopus laevis *and retrieving their potential full length clones was not available before. Moreover, our implementation is specifically designed and focused on relating *Xenopus *sequence data to the major model organisms. Thus, one can search for the *Xenopus *homologue directly using the human or mouse protein.

## Construction and Content

### Sequence sources and cleanup

350,468 Sequences were downloaded from GenBank release 138 and stored in a relational database using the open source ORDBMS PostgreSQL. The following divisions were included: Vertebrate Sequences (VRT, 5,506 sequences), EST (344,747 sequences) and High Throughput cDNA (HTC, 215 sequences). 228,496 sequences were annotated as 5' ESTs and 116,122 as 3' ESTs. 245,415 different cDNA clones were represented in the data set, out of which 92,463 had both 5' and 3' sequences. Entries annotated as being genomic sequences were excluded from the analysis. To enhance the usability and search capabilities of the database, complete GenBank entries were incorporated. Annotations including but not limited to library source, tissue type, cell type and developmental stage were extracted directly from GenBank entries (feature: source, qualifiers: clone_lib, tissue_type, cell_type and dev_stage). Unfortunately, the sequences are not very well annotated in GenBank. 34% of the sequences do not have a tissue type assigned and 36% have no developmental stage information. Distributions of tissue types, developmental stages and clone libraries are shown in supplemental files [see additional files 2, 3 and 4 respectively].

197,888 ESTs (57.4% of the EST sequences) had information about high quality start or end of sequencing reads. This information was used to trim sequences according to high quality regions to insure best sequence quality. Vector sequence was downloaded from GenBank and VectorDB [38] and the sequence masked using the program Vmatch [39] developed by Stefan Kurtz. Vmatch is based on a novel sequence index (enhanced suffix arrays, [32-34]), allowing for the rapid identification of similarities in large sequence sets. ESTs were trimmed to eliminate vector sequence located at either the 5' or 3' end (6678 ESTs, 1.9% of total sequence set). In some cases, additional non vector sequence preceded or followed known vector sequence. If such non-vector sequence was less than 20 bases long, it was trimmed from the EST together with the vector sequence. ESTs that had vector sequences left after trimming were discarded completely. Repetitive elements were obtained from Repbase [40] and GenBank and masked using RepeatMasker [41]. In addition, if hits against ribosomal RNA and mitochondrial sequences were found in the downloaded sequence set, the corresponding sequences were removed. The availability of complete mitochondrial genomic and ribosomal sequences makes the inclusion of these sequences unnecessary while masking was performed to minimize possible clustering errors arising from these common sequences. Sequences that had less than 100 consecutive bases left after cleanup were discarded completely (21,039 sequences, 6.0%). The resulting sequence set consisted of 317,242 sequences (90.5%) with an average length of 536 bases (see Table 1).

**Table 1:** Summary of *Xenopus *EST cleanup and clustering.

Total number of ESTs and cDNAs	350,468
Number of distinct clones	245,415
Number of good sequences	317,242
Average trimmed EST length (bp)	536
Number of 3' EST sequences	116,122
Number of 5' EST sequences	228,496
Clones with 5' and 3' sequences	92,463
Number of clusters	25,971
Number of singletons	40,877
Number of CAP3 contigs	31,353
Number of CAP3 singletons	4,801
Average CAP3 contig length (bp)	1,045
Max. cluster size (no. of ESTs)	6,332
Average cluster size (no. of ESTs)	10.6
**Cluster sizes:**	**# EST**
4,097 – 8,192	1
2,049 – 4,096	1
1,025 – 2,048	2
513 – 1,024	15
257 – 512	35
129 – 256	116
65 – 128	414
33 – 64	973
17 – 32	1,755
9 – 16	2,974
5 – 8	4,571
3 – 4	6,444
2	8,670

### Clustering and assembly of tentative contig sequences

The cleaned *X. laevis *EST sequence set was grouped into gene specific clusters using Vmatch. Vmatch preprocesses the EST sequences into an index structure: an enhanced suffix array. This data structure has been shown to be as powerful as suffix trees, with the advantage of a reduced space requirement and reduced processing time. Further on, enhanced suffix arrays have been shown to be superior to other matching tools for a variety of applications [33,42,43]. For a detailed introduction of enhanced suffix arrays see Abouelhoda et al. [34]. Briefly, the index efficiently represents all substrings of the sequences and allows the solution of matching tasks, in time independent of the size of the index (unlike BLAST). Vmatch was chosen for the following reasons: (1) At first, there was no clustering tool available which could handle large data sets efficiently, and which was documented well enough to allow a detailed replication and evaluation of existing clusters. (2) Second, Vmatch identifies similarities between sequences rapidly, and it provides additional options to cluster a set of sequences based on these matches. Furthermore, the Vmatch output provides information about how the clusters were derived. Due to the efficiency of Vmatch, we were able to perform the clustering for a wide variety of parameters on the complete sequence set (see below). This allowed us to study the effect of the parameter choice on the clustering. Moreover, in the future, the efficiency will allow us to more frequently update the data set. A longer term goal of the project is to generate a data set that maintains the different alleles in this pseudotetraploid animal as separate entries. The clustering approach has been integrated into an analysis pipeline which can be applied to other organisms that often receive less attention from the bioinformatics community.

The database sequences were clustered according to the matches found in a self comparison of the index. Initially each database sequence is put into its own cluster. Then all pairs of matches are generated and each pair is evaluated to possibly form single linkage clusters. To identify matching sequences, Vmatch first computes all maximal exact matches of a given minimal length (seeds) between all sequences. These seeds are extended in both directions allowing for matches, mismatches, insertions, and deletions using the X-Drop alignment strategy as described previously. This greedy alignment strategy was developed for comparing highly similar DNA sequences that differ only by sequencing errors, or by equivalent errors from other sources [44].

In an attempt to objectively define appropriate clustering criteria, we took advantage of the speed of the Vmatch clustering approach to systematically vary the relevant parameters (overlap length, % identity, seedlength and X-drop value). It was hypothesized that the 'correct' parameters would be revealed as an abrupt change in the curve on the resulting graph. An example of such an analysis showing the effect of varying the overlap length and % identity is presented in supplemental materials [see additional file 1]. Here a number of conclusions become apparent. First, at this level of resolution (~30 independent clusterings), a distinct point indicating the 'correct' parameter does not become readily apparent. Second, the collapse of the cluster set to few clusters containing every larger numbers of individual sequences serves as a reminder that all sequences (regardless of species) can be considered part of a single cluster. Finally, as the length overlap decreased, we observed the formation of 'superclusters' containing >10,000 sequences clearly derived from multiple gene families. These problem of 'superclusters' diminished at an overlap length of ~135 (data not shown, and not apparent in additional file 1). These clusters appear to be due to the presence of undefined repetitive elements, chimeric sequences and possibly transposed elements. Studies on the nature of the clustered sequences and the effects of parameter variation are ongoing.

For the current data set, we tried to select parameters which mimic the parameters that were probably used for generating the UniGene clusters. Unfortunately, the algorithm used for constructing the UniGene clusters is not sufficiently documented to allow complete reproduction. We selected parameters designed to produce a stringent clustering of the available sequences. For the described data set, sequences were clustered when a pairwise match of at least 150 nucleotides and 98% identity was found (seedlength = 33, X-Drop = 3). The construction of the enhanced suffix array took 33 minutes on a SUN UltraSparc III (900 MHz) CPU. Clustering took another 17 minutes. This resulted in 25,971 clusters containing 276,365 sequences (87.11% of the input set) and 40,877 singletons (12.89%). The average cluster size was 10.6 (std. dev 51.8) sequences. The distribution of cluster sizes is shown in Table 1. 22,834 clusters were composed of ESTs only, 61 clusters of mRNA sequences (VRT and HTC divisions) only and 3,076 clusters of both mRNAs and ESTs. Among the singletons are 4262 sequences which contain less than 150 nt (after sequence cleanup described above) and would therefore be incapable of being joined in a cluster. Less than 25% of these sequences have a significant match against NR database and less than 2% of the sequences match full length cDNA criteria described below.

Next, a consensus sequence was generated for each cluster using CAP3 [28]. The aim of this approach was to both refine the number of clusters and to improve the overall sequence quality. This latter aim simplifies the design of oligonucleotide probes. The 25,971 clusters produced 31,353 tentative contig (TC) sequences (avg. length: 1,045 bp, std. dev: 729 bp) and 4,801 singlets (avg. length: 664 bp, std. dev: 424 bp). The longest TC was 13,130 bp (DNA-dependent protein kinase catalytic subunit, accession: [Genbank:AB016434]), while the smallest TC was 154 bases long. Here, it became obvious that CAP3 is a genome assembly program not designed to assemble EST clusters containing potential splice variants: CAP3 assembly subsequently split a fraction of the clusters into separate contigs and singletons. On average, a cluster was split into 1.2 (std. dev 3.0) TCs and 1.8 (std. dev 11.3) singlets by CAP3. As illustrated in Table 1, the average length of the sequences increased from 536 bp (average for input ESTs) to 1,045 bp (average for CAP3 contig sequences) which was lower than the average length for previously characterized *Xenopus *full length sequences (sequences selected as full length by XGC had an average length of 2,115 bp).

There are many genes whose transcript is significant longer than 2× the current state of the art sequencing run of ~1000 bp. This means that 5' and 3' sequences derived from a >2 kb transcript are unable to be joined without sequence from incomplete cDNA clones which provide a source of nested deletions. Sequences from both ends can be linked by annotation, and this has been done by a variety of clustering approaches including NCBI UniGene which uses a double linkage rule. Non-overlapping 5' and 3' ESTs are assigned to the same cluster if clone IDs are found that link at least two 5' ends from one cluster with at least two 3' ends from another cluster and the two clusters are merged. We have examined the effect of double linkage joining using the clone annotation. In this analysis, 17,588 clusters were stable and the total number of clusters was reduced from 25,971 to 21,249. Most of the joined clusters (3,122) were created from two clusters while three clusters were combined 456 times. While the number of clusters is decreased by this joining, our overall analysis is not affected. Potential full length clones selected as part of the *P5P *group (see below) are also unaffected by annotation linkage. We provide the identity of clusters 'linked by annotation' as part of the XenDB output.

### Sequence analysis

We have performed a variety of sequence comparisons at the protein level including translation analysis. The sequences of cluster TCs and all singletons were subject to extensive BLASTX [45] and FASTY [46] homology searches vs. the non-redundant protein database (NR) from NCBI and the proteomes of five major model organisms using the high throughput analysis pipeline of the Genlight system [47] Proteome sets for *H. sapiens*, *M. musculus *and *R. norvegicus *were obtained from the *International Protein Index *[48,49]. The IPI provides a top-level guide to the main databases: Swiss-Prot, TrEMBL, RefSeq and Ensembl. It curates minimally redundant yet maximally complete sets of the indexed organisms. *C. elegans *and *D. melanogaster *protein sequences were retrieved from the UniProt database [50]. UniProt proteome sets are solely derived from Swiss-Prot and TrEMBL entries. Additionally, all available protein sequences for *X. laevis *and *X. tropicalis *were extracted from GenBank. additional file 5 provides an overview of the downloaded data sets. Performing separate comparisons allows a search for matching sequences based on the identity of any gene known from each species as well as query for genes which have matches in some but not all databases. We believe that this will aid in the discovery and analysis of conserved and unique genes. In addition to these databases, we have included BLASTX searches in the KOG database and have used the results to functionally classify the *Xenopus *sequences. All sequences resulting from the clustering and assembly processes were compared to these protein sets using BLASTX with an E-value cutoff of 1.0e^-6^. ESTs are often of low sequence quality, and sequencing errors can still exist in the assembled TC sequences. Therefore, all analyses against the protein databases were also done using FASTY (E-value cutoff: 1.0e^-6^) a version of FASTA that compares a DNA sequence to a protein sequence database, translates the DNA sequence in three forward (or reverse) frames and allows (in contrast to BLASTX) for frame shifts, maximizing the length of the resulting alignments.

### Identification of chimeric sequences

**Figure 3:**
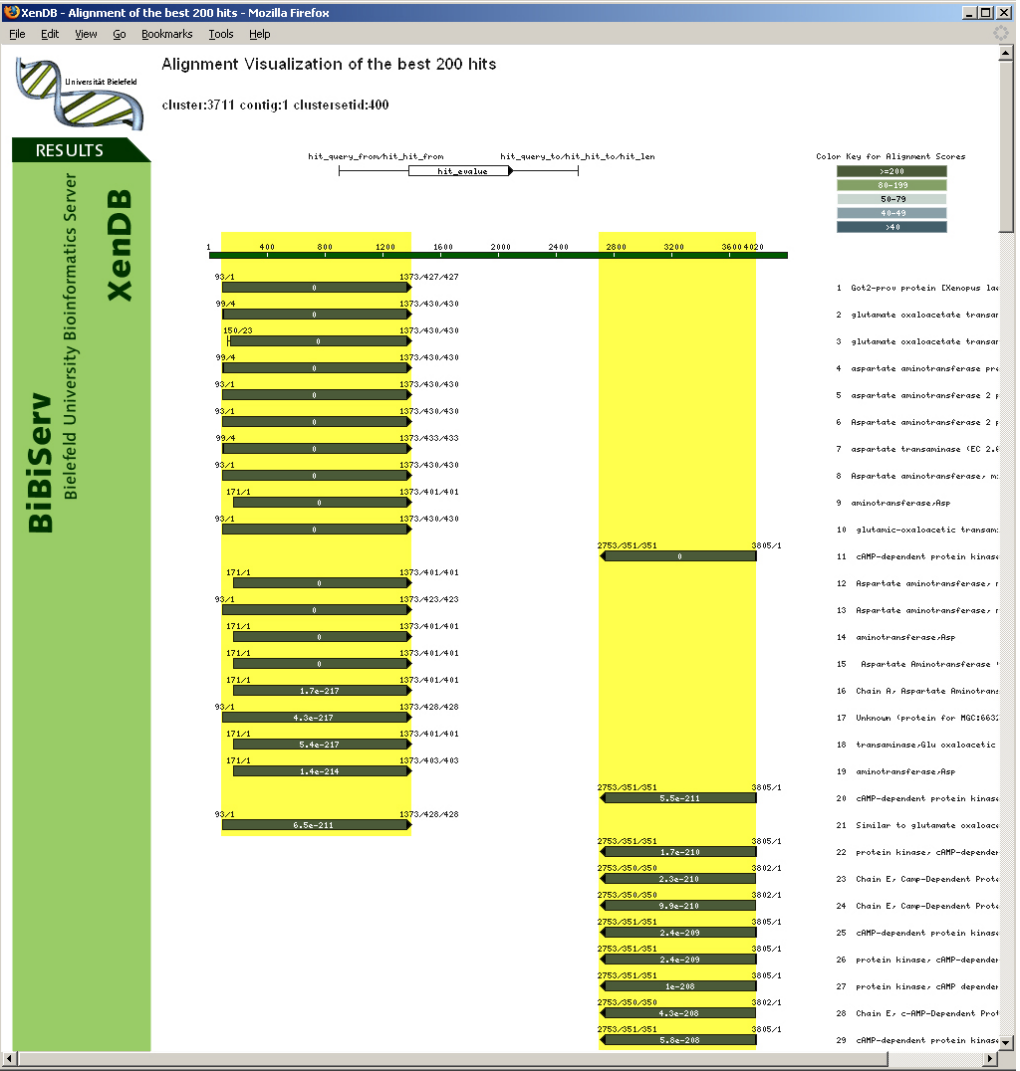
Identification of chimeric TCs: Matches of at least 100 bp in length were mapped back to the TC sequences to identify the regions that are covered by a match (yellow boxes). If two matches overlap, the region will be extended accordingly. If after the mapping two clearly separated regions remain as shown here, the TC is flagged as potential chimera.

A significant issue in EST clustering methods is the presence of chimeric sequence which inappropriately joins unrelated genes into a single cluster. While the number of chimeric sequences is estimated at less than 1% [51,52], their presence has disproportionate effects on the clustering outcome. To identify potential chimeric sequences, we analyzed the FASTY hits in the protein NR database and applied the following simple procedure: Matches of at least 100 bp in length were mapped back to the TC sequences to identify the regions that are covered by a match. If two matches overlap, the region will be extended accordingly. If after the mapping two clearly separated regions remain, the TC is flagged as potential chimera (see Figure 3).

Examination of the identified chimeric sequences reveals three major classes. In the first, two distinct FASTY hits can be identified which do not overlap and are in opposite orientation. In the second, the second identified FASTY hit matches retroviral or transposable element related sequences. This suggests the possibility that these may reflect real transcripts in which a mobile element has been inserted into the genome. A close evaluation of such sequences may provide some insights into the evolutionary history of various populations of *Xenopus*. The final class of potential chimeric sequences identified contains short predicted or hypothetical proteins. This class may in fact not be chimeric at all but may reflect errors in protein coding prediction methods.

The described procedure identified 113 potential chimeric TCs (0.3% of the 33,034 sequences with matches against the protein NR database), which are flagged in the database as such. We do not eliminate these potential chimeras, as they don't significantly affect the results of the sequence analyses done later on, which are mainly based on the best hit only. In fact, the analysis underestimates the number of full length sequences, as some chimeras cover two full length protein matches. A complete identification of chimeric sequences is practically impossible without a comparison to the underlying genome sequence. And even then, polycistronic transcripts which may exist cannot be separated from chimeras perfectly [53].

### Definitions

In the subsequent analyses we were interested in three kinds of information: (1) Full Length Orf containing COntigs (FLOCOs), (2) Full Length Insert containing CLones (FLICLs), and (3) Predicted 5' (P5P) sequences. The result of the clustering and CAP3 analysis generates a set of tentative contig sequences (TC). *FLOCOs *are defined as TC sequences that have an (almost) full length hit against a known protein. These *sequences *are especially useful for gene identification. Full length insert containing clones, *FLICLs*, were predicted. Such *clones *are distinguished by sequence homologies corresponding to the amino terminal part of a protein but are not restricted at the carboxy-terminus. These sequences are derived from clones which are predicted to carry a full length insert (see below), though the full length sequence has not been determined, usually because of single pass EST sequencing from the 5' end. Finally, we identified sequences that we call P5P for which sequence similarity did not extend through the amino-terminal end of the protein but whose length was sufficient to include a full length coding sequence of a similarly sized protein.

### Identification of Full Length Orf containing COntigs (FLOCOs)

**Figure 1:**
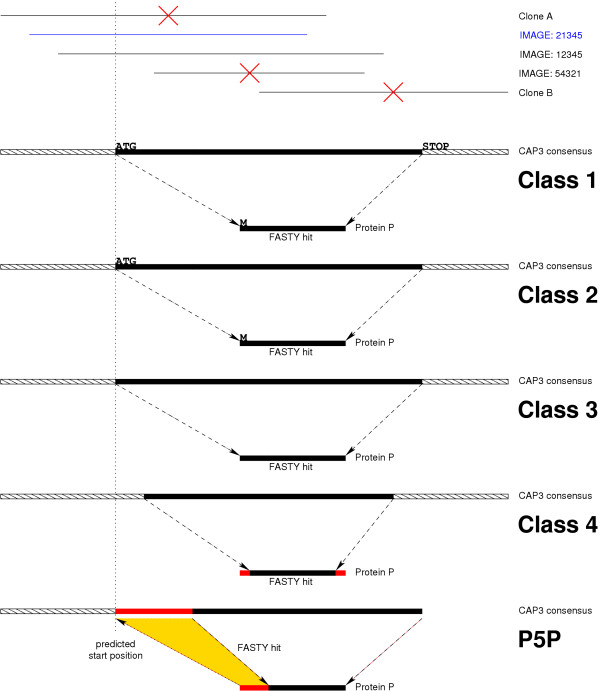
Full length clone selection (top) and TC categories (bottom). ESTs derived from different clones were clustered and assembled. The CAP3 contig was compared to protein databases using BLASTX and FASTY and hits categorized in 4 categories. Class 1 hits had to match the whole protein sequence and start with an ATG in the TC and M in the protein and the hit had to end at a STOP codon. Class 2 hits had to match the whole protein sequence, start with an ATG in the TC and M in the protein. Class 3 had to match the full protein sequence (without further restrictions), class 4 had to cover the protein over almost its full length, allowing the match to start or end maximal 10 ten amino acids after/before the start or end of the protein. Predicted 5' TCs (P5P) had to have enough sequence to fill up the missing 5' end of the protein sequence. Clone selection: Clone A and B were discarded because of missing IMAGE id. Clone 54321 does not span 5' end of protein match. Clone 21345 was selected as most 5' clone fulfilling the requirements.

We were especially interested in full length hits of the TC sequences vs. known proteins. For this purpose, BLASTX and FASTY hits were categorized into four classes, representing the quality of the full length matches (see Figure 1): (1) Matches cover 100% of the sequence of a known protein. Additionally, the matched protein sequence has to begin with the conserved methionine and has to end at a conserved STOP codon. (2) Matches covering 100% of the sequence of a known protein. Additionally, the matched protein sequence has to include the initial methionine. (3) Matches capable of covering 100% of the matched protein sequence with no additional constraints. (4) Matches that cover the protein over almost its full length, allowing the match to start or end maximal ten amino acids after/before the start or end of the protein.

**Figure 2:**
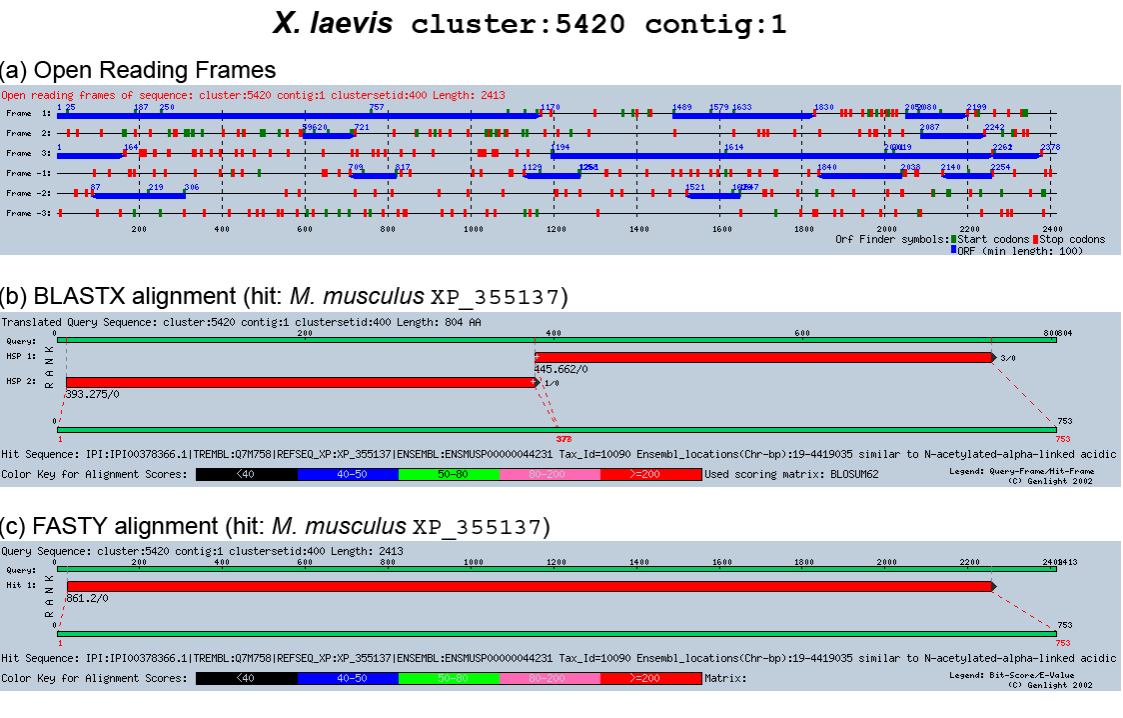
Comparison of a BLASTX alignment with corresponding full length FASTY alignment, as generated by the Genlight system. Blue boxes in (a) indicate open reading frames, green boxes start and red boxes stop codons, respectively. The assembled TC sequence has a frameshift at position 1150 from frame 1 to 3, generating two distinct HSPs in the BLASTX alignment (b). FASTY clearly corrects this frameshift and generates a full length alignment (c).

Table 2 shows the number of identified FLOCOs using BLASTX. 3,942 TCs were Class 1 hits in the non-redundant protein database. As the stringency of the full length definition was relaxed, the number of TCs characterized as full length increases to 5,050 (Class 2), 7,792 (Class 3) and 12,389 (Class 4) TCs respectively. As EST sequences have many sequencing errors, and even the assembly of clusters can not correct all of these, FASTY comparisons were done for the same data set (Table 3). This way, the length of the resulting alignments could be maximized. A comparison of Table 2 and Table 3 shows the effect of frame shift corrections obtained by FASTY. The number of TCs having Class 1 hits could be increased to 5,139 while the less stringent categories increased similarly by an average of 20%. The effect of frameshift correction can clearly be seen in Figure 2. Table 4 and Table 5 show the average lengths of TCs for each of the four categories. Here, the average length of the TCs is 2,210 bp for Class 1 TCs having FASTY matches against X. *laevis*, corresponding very well to already known *Xenopus *proteins. Overall, the average length decreases with lower quality categories as expected, especially for Class 4, where the alignment can miss 20 amino acids on both ends of the matching protein. The only exceptions are Drosophila and *C. elegans*, where the average length increases for Class 4 sequences.

**Table 2:** Number of *X. laevis *TCs with full length BLASTX hits in the non-redundant protein database (NCBI), five model organisms, and available *X. laevis *and *X. tropicalis *proteins, determined by BLASTX. Lower quality categories include sequences from higher, more stringent categories.

**Class**	**Protein NR**	**Human**	**Mouse**	**Rat**	**Fruitfly**	**C. elegans**	**X. laevis**	**X. tropicalis**
**1**	3,942	1,760	1,765	1,455	219	140	2,918	495
**2**	5,050	2,067	2,076	1,736	311	233	3,104	541
**3**	7,792	2,647	2,919	2,592	392	283	3,898	590
**4**	12,389	5,587	5,841	3,078	2,071	1,856	5,024	1,033
**P5P**	15,870	13,942	14,179	13,113	8,425	8,117	9,227	4,334

**Table 3:** Number of *X. laevis *TCs with full length FASTY hits in the non-redundant protein database (NCBI), five model organisms, and available *X. laevis *and *X. tropicalis *proteins, determined by FASTY. Lower quality categories include sequences from higher, more stringent categories.

**Class**	**Protein NR**	**Human**	**Mouse**	**Rat**	**Fruitfly**	**C. elegans**	**X. laevis**	**X. tropicalis**
**1**	5,139	2,347	2,337	1,930	268	190	3,862	660
**2**	6,243	2,692	2,671	2,248	383	296	4,119	721
**3**	9,576	3,528	3,774	3,374	473	357	4,967	796
**4**	14,094	6,467	6,701	6,341	2,249	1,918	5,701	1,241
**P5P**	15,651	13,578	13,954	13,085	8,108	7,746	9,055	4,159

**Table 4:** Average length of *X. laevis *TCs for different BLASTX full length TC categories.

**Class**	**Protein NR**	**Human**	**Mouse**	**Rat**	**Fruitfly**	**C. elegans**	**X. laevis**	**X. tropicalis**
**1**	1984	1835	1805	1788	1620	1541	2171	1743
**2**	1831	1806	1776	1775	1541	1391	2120	1697
**3**	1630	1813	1775	1834	1560	1429	1981	1693
**4**	1393	1680	1675	496	1638	1640	1879	1660

**Table 5:** Average length of *X. laevis *TCs for different FLASTY full length TC categories.

**Class**	**Protein NR**	**Human**	**Mouse**	**Rat**	**Fruitfly**	**C. elegans**	**X. laevis**	**X. tropicalis**
**1**	2007	1888	1859	1843	1659	1575	2210	1807
**2**	1837	1856	1821	1819	1563	1440	2152	1774
**3**	1553	1790	1772	1804	1569	1441	2019	1768
**4**	1329	1683	1673	1664	1611	1563	1910	1703

Comparing the numbers of full length sequences in Table 2 and Table 3, the matches in human, mouse, rat and *X. laevis *are in general agreement (2619 full length sequences for Class 1 on average). What is striking is the deviation of both the number of full length TCs as well as the average length of TCs having matches against Drosophila and *C. elegans*: only 268 and 190 full length sequences with average lengths of 1659 and 1575 bp for Drosophila and *C. elegans *in Class 1, respectively. Only within the Class 4 category there are 2,249 and 1,918 TCs with average lengths of 1,611 bp and 1,563 bp, respectively. A possible explanation for this difference is the divergence of the vertebrate species from these invertebrate model systems.

### Selection of putative Full Length Insert containing CLones (FLICLs)

Often, biologists are interested in identifying a full length clone for further study and this desire has been met by the establishment of a number of the Gene Collections (the Mammalian Gene Collection [54], the *Xenopus *Gene Collection [55] and the Zebrafish Gene Collection [56]). We have extended our analysis described above to select potential full length insert containing clones (FLICLs) that are available through the IMAGE consortium and provide a simple yet powerful search tool to rapidly match homologous genes of interest to their *Xenopus *counterparts. The Gene Collections are an NIH initiative that supports the production of cDNA libraries, clones and 5'/3' sequences to provide a set of full-length (ORF) sequences and cDNA clones of expressed genes for a variety of model systems.

Since the average length of the characterized full length vertebrate protein is 1,400 bases and the average sequence length of a TC is 1,045 bases, many sequences which are full length will not be detected by the previous approach and will contain sequence gaps of approximately 350 bases. To identify additional clones that potentially carry a full length insert, we queried the database for sequence matches which were sufficiently long to include the start methionine but which did not have sufficient homology to be detected by the previous methods Thus, a sequence with a query start position (Start_q_) which is greater than the subject start site (Start_s_) is potentially a full length open reading frame (hereafter referred to as *P5P, predicted 5 prime*). Clearly, the value of such a prediction decreases as the values of Start_q _increases and the predictive value increases with lower values of Start_s_. Full length clones predicted by this method are subject to 3' truncations due to mispriming in poly(A) rich regions rather than at the polyA tail. Such regions would be characterized by the presence of the amino acid lysine (codons AAA, AAG) or asparagine (codons AAU, AAC).

**Figure 4:**
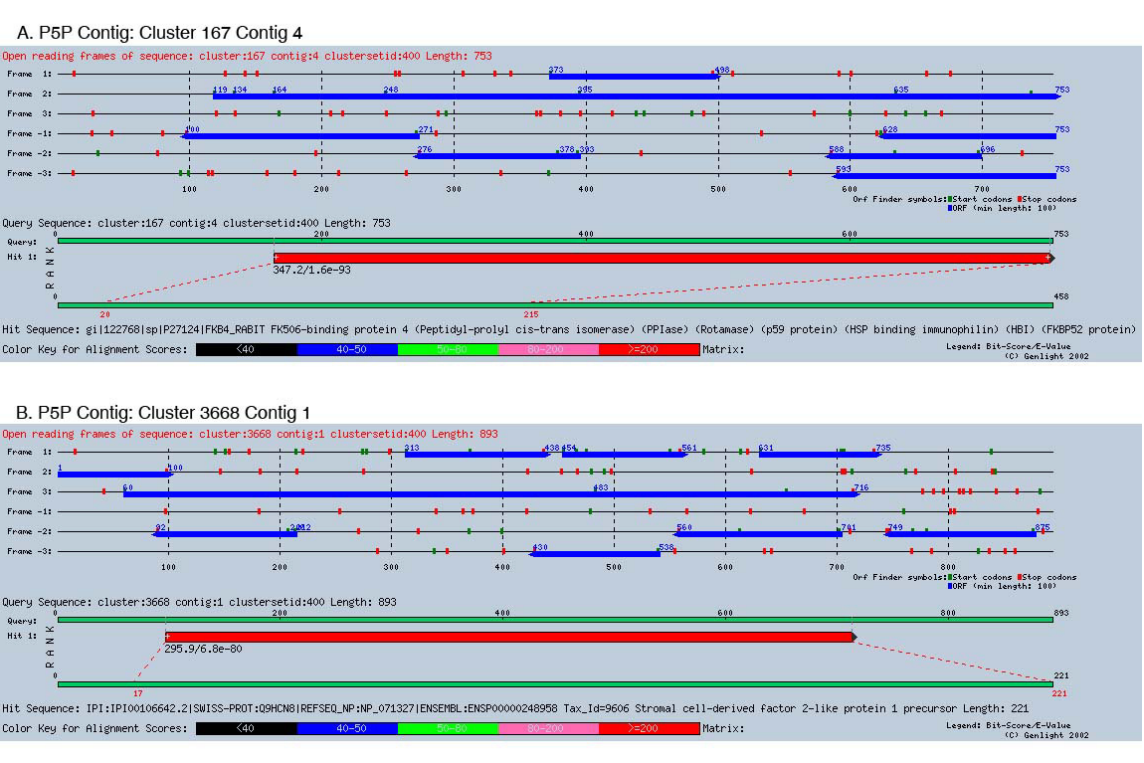
Two examples of TCs derived from clones predicted to have a full length insert (P5P). The start positions in the hit suggest that the unmatched amino-terminal protein sequence is not well conserved between X. *laevis *and the matched organisms, here rabbit (top) and human (bottom), but the open reading frames (blue boxes) indicate that the clones the sequences were derived from do actually contain a full length insert. (Screenshots of the results were generated by the Genlight system.)

Best FASTY hits were extracted for TCs from all four full length categories as well as the P5P categories as described above. For TCs matching these categories, the most 5' EST contributing to the CAP3 contig sequence was selected. In addition, the selected clone had to span the amino-terminal end of the FASTY protein match. Finally, to ensure the ready availability of the clones and therefore the utility of the analysis, the selected clone had to be available through the IMAGE consortium. See Figure 1 for an illustration of 5' clone selection. The P5P criteria selected 15,651 potential full length insert containing clones out of which 10,500 are distinct IMAGE clones, which represents an additional 1,557 sequences compared to Class 4. Two examples of such predicted protein coding sequences are presented in Figure 4. We have mapped these clones to 7,782 distinct clusters. To assess the quality of the FL prediction method, we compared our set to the IMAGE clone set selected by the *Xenopus *Gene Collection (XGC, [55]) for full length sequencing. As of April 2004 the XGC had selected 10,482 IMAGE clones for sequencing. Our analysis selected 3,152 IMAGE clones that were identical to clones selected by the XGC. Of the remaining 7,348 clones from our set, 4,866 selected IMAGE clones were found in an identical cluster as 4,465 XGC selected clones (note that some of these clones are in the same cluster). In addition, 1,154 XGC clones did not have sequence available to be included in our analysis. The remaining 1,711 IMAGE clones selected for sequencing by XGC are not found in our predicted set while 2,482 clones were unique to our set. In an effort to examine why the 1,711 sequences selected for sequencing were not identified as full length, we compared the start_q _and start_s _values as described above. Using the P5P prediction criteria described above, we identify 107 XGC selected IMAGE clones that we predict are not full length but have an alternative clone which we predict is full length. Though final confirmation of the results requires additional sequencing, our method appears to be successful at identifying full length sequences and distinguishing non-full length sequences identified by an independent method. The FL clones are labeled in the XenDB web interface (see below), allowing a rapid identification of potential FL clones for a gene of interest.

Due to the large number of sequences, we are unable to examine each sequence individually. Since the analysis depends on the overall degree of conservation among the sequences, such an approach will not be as successful on weakly conserved genes. In general, it seems likely that decreasing e-values correspond to higher quality predictions. On a global basis, the results need to be carefully considered, as an independent assessment of the distribution of conservation among the ensemble of sequences is not available.

### Gene Ontology prediction and Functional Classification

The Gene Ontology (GO) project [57] is an ongoing international collaborative effort to generate consistent descriptions of gene products using a set of three controlled vocabularies or ontologies: biological processes, cellular components, and molecular functions. The GO vocabulary allows consistent searching of databases using uniform queries. The availability of such vocabularies can be critical to the interpretation of high through put approaches such as microarrays. Based on FASTY homologies with both mouse and human sequence, we have mapped GO annotations to the *Xenopus *sequences. Of the 30,683 TCs with matches to mouse (29,971) or human IPI sequences (29,963), 19,721 TCs have been assigned putative GO annotations. Among the 10,500 potential full length ORF containing IMAGE clones, 6,886 have been assigned GO annotations.

The non-redundant *X. laevis *data set was then classified based on their homology to known proteins from the KOG [58] database (BLASTX 1.0e^-5 ^E-value cutoff, best hit selection). KOGS are *euKaryotic clusters of Orthologous Groups*. KOG includes proteins from 7 eukaryotic genomes: *C. elegans*, *D. melanogaster*, *H. sapiens*, *A. thaliana*, *S. cerevisiae*, *S. pombe*, *E. cuniculi*.17,624 sequences (67.3%) had a hit against the KOG database and could be assigned a functional category.

### Identification of conserved genes not found in major model organisms

To identify additional genes within the dataset that are not found by comparison to protein sets of the major model organisms and to assess the extent of diverged or non conserved sequences, open reading frames of 600 nucleotides or longer were selected from the clustered data set for analysis. 219 sequences that did not have any hit in the previous analyses were identified (188 TCs representing 178 clusters and 31 singlets). We further restricted the number of sequences by re-running the BLASTX and FASTY analysis with E-value cutoffs of 0.01. 111 sequences (91 TCs representing 87 clusters consisting of an average of 6 ESTs per cluster and 19 singlets) without any significant similarity in protein databases could be identified and these were examined by TBLASTN against the human, mouse and 'others' EST databases (22.7 million sequences total). Signal peptides were identified by SignalP [59] as well as transmembrane domains by TMHMM [60,61]. Results are presented in Table 6. The analysis identified 46 sequences with similarity to other organisms (E<0.01) with 11 sequences matching chicken (*Gallus gallus*), 10 sequences matching zebrafish (*Danio rerio*) and 6 sequences matching the rainbow trout (*Oncorhynchus mykiss*). Three of the sequences matched human sequences with less significance than the cutoff used above (i.e. 1.0e^-6^). Among the sequences with highly significant BLAST hits were two matches to the eastern tiger salamander (*Ambystoma tigrinum tigrinum*) and one to the rainbow trout (*Oncorhynchus mykiss*). A surprising match was to barley (*Hordeum vulgare*, E = 9.0e^-35^) which was the only plant represented among these hits. The remaining 65 sequences did not have significant homology to existing public database sequences. For 7 sequences both signal peptide cleavage sites and transmembrane domains could be identified. Another 15 sequences had either a signal peptide cleavage site or a transmembrane domain. These 22 sequences are potentially novel membrane proteins.

**Table 6:** *Xenopus *Long Open Reading Frames (>= 600 nt) without homology to major model organism protein sequences. ORF sequences were compared to all available EST data using TBLASTN. The 46 sequences shown here have homologies to ESTs from other organisms (E < 0.01). For each TC, the number of ESTs in the TC and the accession, SignalP and TMHMM results, and description and E-value of the best hit is shown. Additionally (not shown here), both signal peptides and transmembrane domains could be predicted in: clSignal peptides only in: cl4857_sin8, cl11312_sin2, cl11866_ctg2, cl14117_ctg1, cl16548_ctg1, cl19372_ctg2; Transmembrane domains only in: cl3994_ctg1, vimsin144578, cl18799_ctg1, cl18978_ctg1, cl18978_ctg2, cl25690_ctg1, cl23256_ctg1.

**Contig/ORF**	**#ESTs**	**Accession**	**SignalP**	**TM**	**Description (best hit)**	**E-value**
cl9703_ctg1_1	53	CN060851			Ambystoma tigrinum tigrinum	5.00E-112
cl15798_ctg1_1	4	CN061938			Ambystoma tigrinum tigrinum	6.00E-53
cl9914_ctg1_1	11	BX864357			Oncorhynchus mykiss	4.00E-45
vmsin143901_1	1	CK600275			Rattus norvegicus	4.00E-43
cl10823_ctg1_1	3	CA471690			Danio rerio	5.00E-39
cl2563_ctg2_1	10	AV913994			Hordeum vulgare subsp. Vulgare	9.00E-35
cl1723_ctg1_1	12	BU129000			Gallus gallus	2.00E-34
vmsin213651_1	1	CD218114			Gallus gallus	7.00E-30
cl15560_ctg1_1	3	CK871392			Danio rerio	2.00E-24
cl10197_ctg1_1	3	CA975598			Danio rerio	2.00E-22
cl11603_ctg1_1	4	AJ456928			Gallus gallus	7.00E-22
cl2506_ctg1_1	7	BJ494402			Oryzias latipes	3.00E-19
vmsin144578_1	1	BU241764			Gallus gallus	1.00E-17
cl24411_ctg1_1	2	BW379961			Ciona intestinalis	2.00E-16
cl25096_ctg1_1	2	BX269216			Gallus gallus	5.00E-16
vmsin117573_1	1	BU114361			Gallus gallus	3.00E-14
vmsin141365_1	1	BI385350			Amphioxus Branchiostoma fl.	3.00E-14
vmsin275700_1	1	CN024469			Danio rerio	4.00E-14
cl5895_ctg1_1	12	BX870166			Oncorhynchus mykiss	6.00E-14
cl18998_ctg1_1	2	BW156550			Ciona intestinalis	8.00E-13
cl5042_ctg1_1	14	CN316430			Danio rerio	3.00E-12
cl9402_ctg2_1	2	AJ448952			Gallus gallus	2.00E-11
cl19097_ctg1_1	4	CN023422	yes		Danio rerio	2.00E-09
cl4943_ctg1_1	4	AJ450094			Gallus gallus	5.00E-09
cl19576_ctg1_1	2	BX862425			Oncorhynchus mykiss	4.00E-08
vmsin9176_1	1	CO051215			Leucoraja erinacea	1.00E-07
cl5371_ctg1_1	9	CD295994			Strongylocentrotus purpuratus	5.00E-07
cl10375_ctg1_1	9	BU133150	yes		Gallus gallus	7.00E-07
cl3127_ctg2_1	19	CF577195			Saccharum sp.	2.00E-05
vmsin5140_1	1	BM265659			Danio rerio	6.00E-05
cl15473_ctg1_1	8	CN502421			Danio rerio	7.00E-05
cl3097_ctg2_1	30	CA374396			Oncorhynchus mykiss	8.00E-05
cl9923_ctg1_1	2	DAA01768			Lytechinus variegatus	1.00E-04
cl15340_ctg1_1	14	CN180033	yes	1	Danio rerio	3.00E-04
cl11246_ctg1_1	6	BX302229			Oncorhynchus mykiss	5.00E-04
cl4857_ctg3_1	7	BF718744			Homo sapiens	6.00E-04
cl18267_ctg1_1	2	AAS58046			Babesia bovis	0.001
cl5917_ctg1_1	6	CN004343			Canis familiaris	0.002
cl9934_ctg1_1	4	CD740019	yes	2	Gallus gallus	0.002
cl3233_ctg1_3	12	CN506386	yes	5	Danio rerio	0.003
cl22258_ctg1_1	2	BM485921			Gallus gallus	0.004
cl14723_ctg1_1	3	BF037758			Homo sapiens	0.005
cl5206_ctg1_1	9	BG166355			Homo sapiens	0.005
cl5199_ctg2_1	8	BM627372	yes	1	Anopheles gambiae	0.006
vmsin18077_1	1	BX877871	yes	1	Oncorhynchus mykiss	0.007
cl5686_ctg1_1	2	BG783827			Strongylocentrotus purpuratus	0.008

## Utility

### User interface

**Figure 5:**
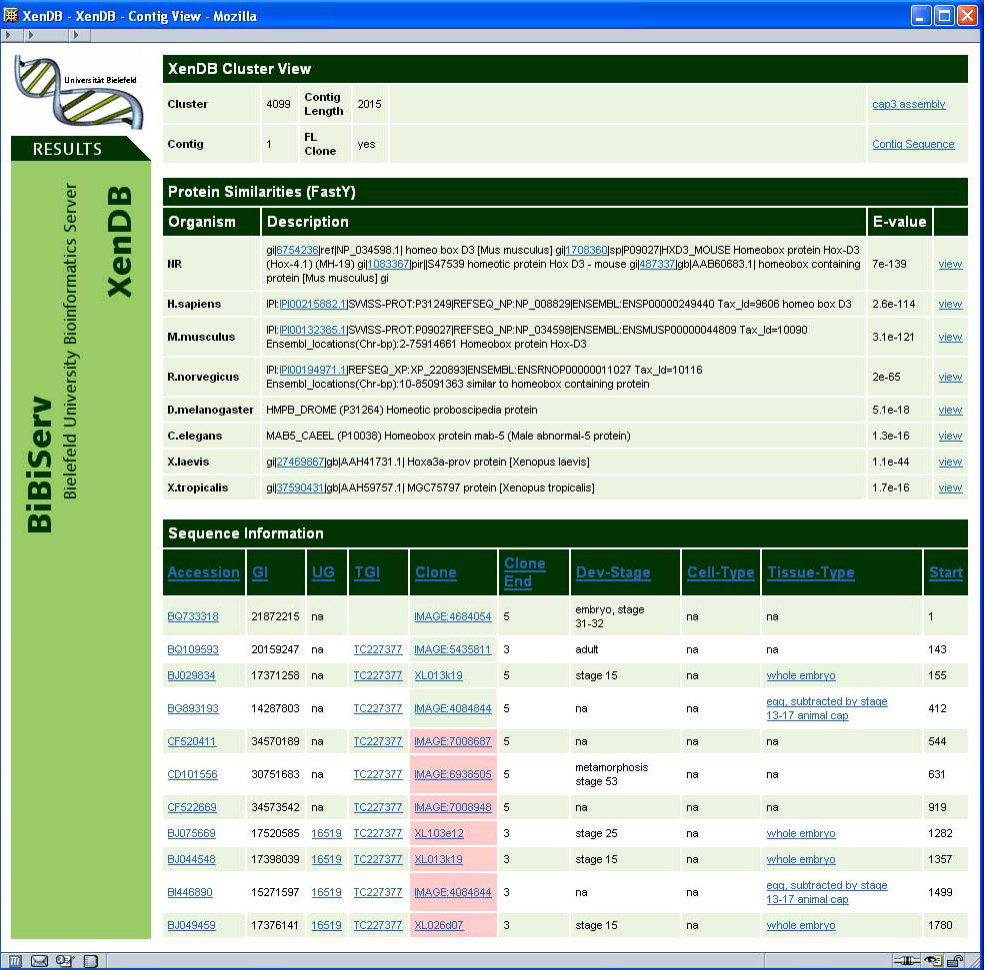
Cluster view of the XenDB Web interface. Best FASTY hits to NR protein database, five model organisms and *Xenopus *proteins are shown on top. Gene Ontologies (GO) are based on best human and mouse IPI hits, functional categories on hits to COG and KOG databases. Below, additional information for each EST in the cluster is shown, such as accession, UniGene and TGI id, clone, cell and tissue type. Clones predicted not to be full length are colored red. Links to CAP3 assembly and TC sequence are provided.

The results of the analyses described above have been incorporated into an SQL database amenable to complex queries. The database can be accessed through a user friendly web based interface (XenDB). XenDB allows individual and batch queries using *Xenopus *accession, GI, and XenDB, UniGene and TIGR cluster IDs. In addition, the user can query the *Xenopus *sequence hits using any protein accession/GI number both singly and in batch mode. This allows a rapid identification of *Xenopus *TCs and their corresponding clones with hits to given protein sequences. The output of various queries displays the matching *Xenopus *cluster(s) and links to a web page as presented in Figure 5. For each cluster, links to the best hit for a number of model organisms are provided as well as links to the assembly result, consensus sequence generated by CAP3, and visual alignments of all FASTY results. GenBank accession numbers for each EST in the cluster and whether the corresponding clone has been identified as full length are provided. Additionally, for each TC the COG and KOG classification, as well as the GO terms are available.

The analysis and database system provides a very powerful tool which will enable the *Xenopus *community to take advantage of a number of technical and experimental advances. We have selected a couple of examples to illustrate possible types of queries. In considering the results, it is important to bear in mind that these examples can be combined to further refine the sequence set. In the first example, we sought to identify all the genes of a known type or class. In the second example, we wished to identify the set of *Xenopus *sequences which best matched a set of genes from another species identified using the CGAP database administered by the National Cancer Institute (NCI) [62,63]. A final example demonstrates the ability of the system to translate results identified by microarray technologies, or other related high throughput technologies, to identify likely *Xenopus *homologues.

### Homeobox gene identification

Homeobox containing proteins are a very important group of transcriptional regulators that play key roles in developmental processes. They can be divided into a 'complex' and a 'dispersed' super class representing the homeotic genes and the large number of homeodomain containing proteins dispersed (and diverged) within the genome [64]. The homeotic (Hox) genes play key roles in the anterior-posterior patterning of both vertebrate and invertebrate embryos and in *Xenopus *are often used as markers of anterior-posterior development. [65-67]. The vertebrate homeotic genes are organized into four clusters arranged in the same order in which they are expressed in the anterior-posterior axis [64]. Of the 39 vertebrate Hox genes, we have identified 28 homologs in *Xenopus laevis*, while 19 are present in the protein database (Table 7). For those sequences not identified, we sought to determine whether they had been identified in the genome of *Xenopus tropicalis*. To do so, we used TBLASTX, provided as a tool on the *Xenopus tropicalis *website [68] to search for the missing sequences. Strong matches were identified for all of the remaining Hox genes except HoxD12. Using the BLASTN tool on the genome site, we confirmed that the gene order was conserved within each scaffold (data not shown). Interestingly, we were unable to identify HoxD12 within the predicted region though both HosxD11 and HoxD13 were recognized.

**Table 7:** Homeobox genes in *X. laevis: *for each HOX gene the corresponding cluster and TC is shown, as well as the most 5' clone in the assembly and the protein accession number, if available. When *X. laevis *genes were not identified, an identifier corresponding *X. tropicalis *sequence is provided.

**IPI Accession**	**Description**	**Xenopus cluster/contig**	**FASTY e-value**	**BLASTX e-value**	**FL Clone**	**Protein Accession**
IPI00027694	HOX-A1.	cluster:4123 contig:1	4.0e-85	1.99E-99	5536792	AAH44984
IPI00012049	HOX-A2.	cluster:7495 contig:1	4.1e-130	7.64E-145	3556495	AAG30508
IPI00012050	HOX-A3.	cluster:10945 contig:1	6.5e-91	2.89E-111	4683538	AAH41731
IPI00020926	HOX-A4.	fgenesh.C_1023000005				
IPI00302291	HOX-A5.	cluster:25739 contig:1	6.9e-44	1.27E-38		
IPI00010742	HOX-A6.	fgenesh.C_1023000003				
IPI00010743	HOX-A7.	cluster:3210 contig:1	5.8e-40	1.17E-64	XL071e19	AAA49753
IPI00010744	HOX-A9.	vm_singlet:264323	1.2e-33	3.48E-29		
IPI00010731	HOX-A10.	fgenesh.C_1487000003				
IPI00010754	HOX-A11.	cluster:6499 contig:1	7.2e-42	Was C11	XL088b06	
IPI00305850	HOX-A13.	vm_singlet:174355	3.8e-57	1.22E-97		
IPI00294724	HOX-B1.	fgenesh.C_2225000001				
IPI00027261	HOX-B2.	fgenesh.C_2225000002				
IPI00027259	HOX-B3.	fgenesh.C_2225000003				
IPI00014540	HOX-B4.	cluster:22503 contig:1	1.2e-27			
IPI00012514	HOX-B5.	vm_singlet:57425	8.5e-35	3.92E-59		
IPI00015075	HOX-B6.	cluster:2339 contig:1	6.2e-42	2.52E-72	XL098k02	
IPI00172584	HOX-B7.	cluster:1985 singlet:1	2.6e-65	8.16E-77	4201615	P04476
IPI00014536	HOX-B8.	cluster:16406 contig:1	2.8e-28	9.90E-43		
IPI00014539	HOX-B9.	cluster:8543 contig:1	4.0e-30	1.05E-50	XL069k06	P31272
IPI00030703	HOX-B10.	cluster:24736 contig:1	5.6e-48	6.95E-74		
IPI00295561	HOX-C4.	fgenesh.C_202000010				
IPI00022893	HOX-C5.	vm_singlet:33065	1.5e-41	6.14E-32		
IPI00015921	HOX-C6.	cluster:9871 singlet:1	4.2e-93	3.16E-109	4202432	P02832
IPI00010756	HOX-C8.	cluster:11257 contig:1	5.2e-95	9.74E-118	XL045l21	AAB71818
IPI00010757	HOX-C9.	fgenesh.C_202000007				
IPI00020947	HOX-C10.	cluster:3243 contig:1	1.3e-51	1.63E-127	4970594	AAO25534
IPI00011610	HOX-C11.	fgenesh.C_202000005				
IPI00010758	HOX-C12.	vm_singlet:240042	2.4e-46	2.75E-22		
IPI00010759	HOX-C13.	cluster:21388 contig:1	2.0e-80	5.86E-89	XL064e01	
IPI00001551	HOX-D1.	cluster:9419 contig:1	2.5e-50	1.68E-65	3475513	AAA49745
IPI00215882	HOX-D3.	cluster:4099 contig:1	2.6e-114	3.48E-121	4684054	
IPI00012390	HOX-D4.	cluster:21685 contig:1	7.1e-67	7.99E-83	5571854	AAQ95789
IPI00008481	HOX-D8.	cluster:11793 contig:1	5.8e-62	2.08E-74	5543040	AAH60408
IPI00292734	HOX-D9.	cluster:13847 contig:1	6.5e-38	5.28E-55	XL045k22	CAC44973
IPI00292735	HOX-D10.	cluster:6503 contig:1	3.8e-135	3.97E-143	4032032	CAC44974
IPI00305856	HOX-D11.	fgenesh.C_1333000003				
IPI00018803	HOX-D12.	missing				
IPI00018806	HOX-D13.	cluster:13386 contig:1	1.7e-93	2.17E-112	3399571	AAO25535

### Homologue identification from the Cancer Genome Anatomy Project (CGAP)

A second example takes advantage of the CGAP database [69] administered by the National Cancer Institute (NCI). This database and resource incorporates a large number of interconnected modules aimed at gene expression in cancer. Among the modules are a Serial Analysis of Gene Expression (SAGE) database [70,71]. The SAGE approach counts polyadenylated transcripts by sequencing a short 14 bp tag at the genes 3'end and is a quantitative method to examine gene expression [70]. Another module is the Digital Gene Expression Displayer (DGED) which distinguishes statistical differences in gene expression between two pools of libraries [72]. Each method generates tables of genes based on a wide variety of selection criteria. As would be expected, the source for the vast majority of the available data comes from either human or mouse thus demanding a tool to cross match the results in *Xenopus*.

For this particular example, we selected a tissue based query (DGED) derived from SAGE data in which we sought a set of genes that might include potential markers for glial or astrocyte fates. For this query, we selected all brain, cortex, cerebellum and spinal cord libraries excluding any libraries derived from cell lines. This yielded 58 potential libraries. From this we selected any library labeled as a glioblastoma for pool A and libraries labeled astrocytoma for pool B while excluding the remaining libraries (which included medulloblastomas, ependymomas, etc.). We did not distinguish between cancer grades. This limited the total number of libraries to six glioblastoma and nine astrocytoma libraries containing 487,197 and 863,610 SAGE tags each, respectively. Submission of the query resulted in the identification of 395 tags with a 2× expression factor and a 0.05 significance factor (default CGAP query values). These 395 tags represented 308 different sequences (180 were >2 fold higher in glioblastoma and 128 were >2 fold higher in astrocytoma) which corresponded to 278 proteins in the public database (115 glioblastoma, 163 astrocytoma) and were matched using the batch GenBank accession module available online in XenDB to 100 and 142 *Xenopus *sequences, respectively. (In the interests of space we have not included the extended table but provide the saved DGED query [see additional file 6] and the two text files [see additional files 7 and 8] that can be uploaded to the XenDB database). The results table includes links to the matching cluster and TC, the e-value and rank and whether a full length clone has been identified. The contig web link leads to additional information including the consensus analysis, the top FASTY hits to five model organisms and links to the *Xenopus *EST sequences in the TC (Figure 5). Among the genes identified are vimentin (15×, P = 0.01) and sox10 (7.6×, P = 0.03), genes previously established as markers of glial and oligodendrocyte fate respectively [73-75] as well as genes downstream of the Notch signalling pathway, known to be important for glia formation [76]. Thus the system developed and presented here allows 'in silico' based tools established for the study and analysis of other organisms, particularly human and mouse, to be easily and rapidly applied to the *Xenopus *model system.

### Homologues of Drosophila eye development genes

In the final example, we take advantage of the database to perform a comparative analysis of microarray expression data. In many instances, the outcome of an array type experiment is a variety of tables listing regulated genes and the associated expression changes. Currently, there are few published *Xenopus *array studies available [77-85] while there exist extensive databases of expression for a variety of model organisms. The NCBI maintains a common database, the Gene Expression Omnibus [86] which contains data from over 15,000 samples including 337 Human, 92 mouse and 12 Drosophila experiments (average 25 samples/experiment). Based on an ongoing interest in eye development, we selected a recent paper by Michaut and co-workers in the Gehring lab which examined gene expression changes induced by ectopic expression of the eyeless gene (ey/Pax-6) in Drosophila imaginal disks [87]. The development of the eye is evolutionarily conserved among both vertebrates and invertebrates [88,89]. Many important insights into eye development have come from studies in Drosophila which has defined a genetic cascade of evolutionarily conserved regulatory factors [90]. One such factor is Pax-6/eyeless which is capable of inducing ectopic eyes on both flies [91] and vertebrates [92]. In the Michaut study, 371 eye-induced genes are detected using two different oligonucleotide based array platforms (Affymetrix and Hoffmann-LaRoche) and 73 are discussed in detail within the text (Michaut et al., Table 1, 2). To identify likely homologues of these genes in *Xenopus*, GenBank accession numbers were obtained from the NCBI Gene Expression Omnibus ([93], accession # GSE271) and used to query the XenDB database to identify 47 potential homologues of the Drosophila Pax6/ey regulated genes and included 32 predicted full length sequences (Table 8). As these sequences are available from commercial sources, they can be readily obtained and tested using the various experimental approaches available to *Xenopus *such as gain of function studies by microinjection.

**Table 8:** *Xenopus *matches to Pax6/ey Regulated Genes identified by Michaut et al.

**#**	**Cluster**	**Ctg**	**FL clone**	**Protein Accession**	**Description**	**E-value**	**DM Rank**	**All Rank**
1	21344	1	YES	AAA19592	Lola protein short isoform	3.90E-10	42	508
2	21344	1	YES	AAA19593	Lola protein long isoform	7.00E-10	67	553
3	22774		NO	AAA21879	atonal protein	1.20E-17	3	21
4	5646	1	YES	AAA28528	fasciclin II	4.30E-28	5	61
5	3838	1	NO	AAA28723	eyes absent	1.80E-118	1	49
6	10868	1	YES	AAB61239	bunched gene product	2.00E-21	6	39
7	BJ063320		NO	AAC46506	Dachshund	1.60E-16	8	44
8	10334	1	YES	AAC47196	Lozenge	2.90E-56	4	77
9	7019	1	YES	AAD38602	scratch	4.40E-35	15	83
10	4763	2	YES	AAD38642	BcDNA.GH11415	2.90E-146	3	15
11	16925	1	YES	AAD38646	BcDNA.GH11973	8.20E-14	1	14
12	18882	1	NO	AAD52845	Pebble	7.40E-62	2	14
13	3666	1	YES	AAF24476	Sticky ch1	1.70E-11	3	56
14	7799	2	YES	AAF48990	CG12238-PA	1.70E-22	3	64
15	19264	1	YES	AAF55415	CG5407-PA	6.30E-198	3	10
16	5529	1	YES	AAF57639	CG15093-PA	2.20E-45	1	24
17	CD327522		NO	AAK06753	roughoid/rhomboid-3	1.10E-29	8	26
18	22774		NO	AAK14073	DNA-binding transcription factor	8.60E-10	11	158
19	1415	445	YES	AAL86442	slamdance	5.50E-70	26	194
20	BU911996		NO	AAN74533	transcription factor fruitless	7.90E-10	28	459
21	CD329851		NO	BAA78210	white protein	2.20E-36	17	54
22	21321	1	YES	CAA33450	glass protein	2.20E-45	21	1739
23	2426	1	YES	CAA38746	neurotactin	2.40E-24	103	706
24	9209	1	YES	CAA52934	Drosophila cyclin E type I	2.50E-56	2	25
25	18485	1	NO	CAA76941	UNC-13 protein	2.70E-165	1	14
26	17438	1	NO	NP_523928	CG7525-PA	8.70E-24	101	1508
27	570	1	YES	NP_524354	CG4236-PA	0	1	17
28	BI349728		NO	NP_573095	CG9170-PA	2.70E-17	1	7
29	1761	1	NO	NP_609033	CG9536-PA	1.20E-21	1	6
30	12008	1	YES	NP_609545	CG14946-PA	9.10E-25	8	63
31	440	2	YES	NP_610108	CG8663-PA	5.70E-17	5	90
32	9019	1	YES	NP_611013	CG11798-PA	1.40E-07	156	2411
33	10147	2	YES	NP_648269	CG5653-PA	1.90E-16	5	48
34	3752	1	YES	NP_649919	CG9427-PA	4.10E-13	1	32
35	20081	1	YES	NP_725617	CG5522-PF	7.10E-49	1	18
36	2636	2	YES	NP_729075	CG10625-	1.70E-28	16	1185
37	8386		YES	O18381	Eyeless protein	3.90E-70	7	75
38	11614	1	YES	P00528	Tyrosine-protein kinase Src64B	4.30E-152	3	150
39	4073	1	NO	P10181	Homeobox protein rough	3.10E-14	13	165
40	919		NO	P20483	String protein (Cdc25-like	3.30E-40	3	43
41	1777	1	YES	P36872	Twins protein (PR55)	0	2	41
42	9517	1	YES	P48554	Ras-related protein Rac2	1.00E-109	1	22
43	7661	1	YES	Q01070	E(spl) mgamma	5.50E-19	5	52
44	7661	1	YES	Q01071	E(spl) mdelta	1.10E-15	7	63
45	4146	2	YES	Q23989	Villin-like protein quail	6.30E-23	9	138
46	10061	1	YES	Q27324	Derailed protein	1.20E-45	23	400
47	14903	1	YES	Q27350	Sine oculis protein	3.90E-87	1	20
**Sequences without significant homology**								
48	O77459	transcription factor Ken			60	NP_651346	CG11849-PA	
49	AAF46666	CG10527-PA			61	Q23997	Chitinase-like protein DS47 precursor	
50	NP_728586	CG9134-PA			62	AAD09748	Gasp precursor	
51	NP_609450	CG17124-PA			63	AAF63503	SP2523	
52	CG140595	Zea mays genomic			64	AAF47412	CG13897-PA	
53	NP_570064	CG10803-PA			65	AAL27368	zinc finger C2H2 protein sequoia	
54	NP_650785	CG5835-PA			66	NP_730444	CG32209: CG32209-PB	
55	AAF51847	CG11370-PA			67	NP_723827	CG18507-PA	
56	AAG46059	SKELETOR			68	NP_611728	CG13532-PA	
57	AAN61340	BcDNA:GH10711			69	NP_651343	CG13651-PA	
58	NP_729183	CG10121-PA			70	NP_995997	CG12605-PA	
59	AAO39528	RE22242p			71	NP_610067	CG9335-PA	

## Discussion

Comparative approaches to important biological problems have resulted in enormous progress in the past decades. The advent of genomic and proteomic approaches has led to a torrent of data in many organisms and has demanded increasingly sophisticated bioinformatic approaches to organize and manage the information. We have developed an integrated information resource with a user-friendly interface powered by an automated clustering pipeline which will allow researchers to take advantage of the wealth of knowledge available in the public domain.

### Comparison to human and mouse

Human and mouse are the best studied vertebrate organisms at the molecular level. In addition to the well publicized genome projects, both have extensive EST collections. This has led to the prediction and characterization of 44,775+ human sequences and 36,182 mouse sequences [94]. As vertebrate development is well conserved, it is important to assess the extent to which the *Xenopus *EST project has identified the known vertebrate genes. At the same time, one would like to identify any genes that are unique to *Xenopus*. Most gene prediction programs rely on homology thus eliminating this approach to unique gene identification. Sequences without significant homology could arise from incomplete sequencing that does not extend into the coding region. Results of the human genome project suggest that this would not be the case for a majority of the sequences analyzed in this report. The average 5' UTR in humans is 240 bp and the 3' UTR is 400 bp [95]. Sequencing reactions with current technologies yield readable sequence of 700 bases on average. Therefore, at least some subset of sequences would yield their protein sequence to analysis. An alternative origin of non-homologous sequences would be unspliced or improperly spliced transcripts. This possibility is also minimized by the utilization of polyA tails for RNA selection and reverse transcription priming using oligo(dT). A final, obvious and expensive approach is to select non-homologous sequences for full length double stranded sequencing. Sequence without errors more easily yields the desired open reading frame in even the simplest bioinformatic programs.

### Sequences without hits

A class of sequences includes those without significant BLAST hits. In our analysis we have used a cutoff e-value of 10e^-6^. This of course is necessarily arbitrary, since as mentioned above it is not known what the exact level of similarity is between any given sequence pair. Based on this value, we remain with 43,753 sequences that neither have a BLASTX nor a FASTY hit to a known model organism sequence. The lack of similarity could be due to significant divergence of the sequence, the lack of an appropriate homologue in the public dataset, sequencing errors inherent in EST data or due to the presence of non-coding, presumably regulatory sequences, in the EST clone set. These unmatched sequences mirror the situation in the UniGene set for both mouse and human with greater than 3 and 4 × 10^6 ^EST sequences in 76,000 and 106,000 clusters respectively while fewer than 25,000 coding sequences have been recognized [21,94,96]. The source of these discrepancies are currently unclear, but may arise from non coding RNA (ncRNA)[97], micro RNA precursors [98], incompletely or unspliced transcripts [99]. In particular, ncRNAs are a likely source for a large fraction of the discrepancy based on estimates of a 10-fold greater number of non-coding transcription units than protein coding genes [100]. It has been estimated that >95% of transcription is non-coding [101]. Much of the analysis and identification of ncRNA relies on the availability of genomic sequence which is currently unavailable for *X. laevis *and incomplete for *X. tropicalis*, the highly homologous diploid species.

### Completeness of Xenopus EST set

We have compared all the *Xenopus *sequences to the human and mouse protein sets to identify conserved proteins. An obvious question is how complete is the *Xenopus *EST set and what percentage of genes have been identified assuming that the vast majority of protein coding sequences have been evolutionarily conserved. Of the ~40,000 sequences in the IPI databases, 9,225 human and 7,664 mouse sequences do not have a strong match (E < 1.0e^-6^). Thus, there is a considerable effort remaining to develop a complete *Xenopus *protein coding set. In the course of our analysis we note the high degree of similarity between the allotetraploid *laevis *and diploid *tropicalis Xenopus *species which depended on the length of the matching sequence. For sequences covering >= 95% of the query, there was an average of 94% identity while the average identity dropped to 91% and 88% as the coverage dropped to 90 and 80% respectively. This conservation may allow sequences from both species to be combined to generate a more complete set.

It is well known that the outcome of clustering methods on a large scale depends on the variety of involved parameters. A systematic comparison between UniGene or TIGR Gene Indices and our results turns out to be extremely difficult, mainly because the underlying sequence sets differ as well due to different sequence cleanup and masking approaches. To maximize the utility and usability of our analysis, we have incorporated UniGene and TGI information into our dataset and provide simple tools for identifying the related UniGene and TGI identifier.

### Future prospects

Both the clustering and consensus generation approaches are very rapid: 50 minutes for clustering on a single 900 MHz SPARC-CPU and a few hours for assembly on a cluster of 20 heterogeneous SPARC-based machines with 450 to 900 MHz. We therefore have achieved the design goal of being able to frequently update this aspect of the analysis. The subsequent comparative sequence analysis requires significantly greater resources and time (several weeks on same cluster of heterogeneous workstations). The analysis described above is performed by various PERL based scripts developed during the course of our analysis which will allow updates and application to other model systems. We are currently working on a tool to compare clusters over time which will allow the sequence analysis described below to be performed on the restricted set of modified/new clusters rather than to the entire ensemble. The effect of CAP3 consensus generation is that a given cluster can be split into several separate TC sequences, usually due to low sequence quality or differences in the UTR regions of the sequences. The UTR end splitting is likely due to the differences between the in-paralogs in this allotetraploid species. We believe that such information will be of value to those researchers interested in a variety of evolutionary questions, examples of which will be discussed below. The difference in ploidy makes *Xenopus laevis *distinct from all of the other organisms for which similar analysis have been performed.

As with all ongoing high throughput sequencing efforts, certain aspects of the results change in proportion to the total number of sequences. As noted above, a complete gene set for *Xenopus *will require additional sequencing. The generation of tetra, octo and dodecaploid species of *Xenopus *between 80 and 10 million years ago [102] offers opportunities in the field of evolutionary biology. For example, comparisons of 3' UTR regions between in-paralogs of *Xenopus laevis *and their counterpart diploid tropical species may improve statistical models of molecular evolution. At the genome level, the potential availability of genome data from the polyploid species may provide insight into questions of chromosome segregation and silencing. The selection of *Xenopus *as a model organism by the NIH  and the establishment of the Trans-NIH *Xenopus *Initiative [103] have directly led to the support of EST and genome sequencing efforts. Among the priorities identified is the establishment and funding of a *Xenopus *Database [104] which will integrate sequence, expression and other *Xenopus *data. We hope to be able to update the results described here on a regular basis and contribute to the community effort.

## Conclusion

One of the primary goals of the effort was to provide a resource of gene-oriented EST clusters and transcript oriented TCs, enriched with various information from heterogeneous sources, that would be of value to the biology community and the *Xenopus *community in particular. Using the XenDB system, the biologist can identify sequences of interest using simple gene name queries, accessions, or gene ontologies. The identified sequences have been mapped to public resources like NCBI's UniGene and TIGR Gene Indices and a consensus sequence prepared. In addition, we have identified publicly available IMAGE clones that maximizes the 5' sequence to provide a full length construct when possible. These clones are available from IMAGE consortium providers.

## Availability and requirements

### Sequence availability, XenDB database and results display

The database and associated files are freely accessible through the XenDB website: . The GenBank accession numbers and FASTA formatted files of the masked and clipped input sequences, as well as the TC sequences and results of the example applications (see below) can be downloaded. Additionally, the list of full length clones is available to researchers interested in performing genome-wide studies. Programs, scripts and database dumps are available from the authors upon request. The XenDB database should be cited with the present publication as a reference.

## List of abbreviations used

EST: Expressed Sequence Tag, ORDBMS: Object Relational Database Managemant System, TC: tentative contig sequence, KOG: clusters of euKaryotic Orthologous Groups, GO: Gene Ontology, VRT: Vertebrate Sequences, HTC: High Throughput cDNA, XGC: *Xenopus *Gene Collection, MGC: Mammalian Gene Collection, ZGC: Zebrafish Gene Collection, FL: Full Length, IPI: International Protein Index, CGAP: Cancer Genome Anatomy Project, DGED: Differential Gene Expression Database, SAGE: Serial Analysis of Gene Expression, ncRNA: non-coding RNA, TGI: TIGR Gene Index

## Authors' contributions

A.S. developed and implemented the Vmatch based clustering pipeline. M.B. contributed his high throughput sequence analysis system Genlight. A.S. and M.B. developed the XenDB database schema, performed the post clustering data analyses and contributed to the manuscript. A.H.B. provided supervision and guidance on the development of the project design goals and the interpretation of analysis output with regard to biological significance. R.G. provided supervision and guidance on the development of the clustering pipeline and provided essential infrastructure. C.R.A. provided advice and guidance on the development of the clustering pipeline, the incorporation of analysis into the database and performed and interpreted the various queries presented and wrote a significant portion of the manuscript.

## Supplementary Material

Additional File 2 Table S1, Distribution of EST sequences in the analysis based on the annotated tissue source for the preparation of the library. (NOTE: annotations are imported directly from GenBank entries and are dependent on the original annotation.) Click here for file

Additional File 3 Table S2: The 20 most abundant developmental stage annotations in the X. *laevis *data set as annotated in GenBank: Distribution of EST sequences in the analysis based on the annotated developmental stage of the source library. (NOTE: annotations are imported directly from GenBank entries and are dependent on the original annotation.) Click here for file

Additional File 4 Table S3: The 30 most abundant Clone Libraries in the X. *laevis *data set as determined by the GenBank annotation. (NOTE: annotations are imported directly from GenBank entries and are dependent on the original annotation.) Click here for file

Additional File 1 Figure S1, Effect of Parameter Variation on EST Clustering: Masked and trimmed EST sequences were clustered using the Vmatch algorithm using different overlap length and percentage identity values. The total number of clusters (blue) and the number of singletons (red) are plotted against the minimal overlap length. Values were plotted at different percentage identities (squares 98%, stars 96%, circles 94%). Click here for file

Additional File 5 Table S4: Sizes of protein sets used for sequence analysis of clustered sequences. Click here for file

Additional File 6 file containing the SAGE database query used in the glioblastoma and astrocytoma analysis. Click here for file

Additional File 7 File containing protein accession numbers of SAGE glioblastoma genes for upload to XenDb system Click here for file

Additional File 8 File containing protein accession numbers of SAGE astrocytoma genes for upload to XenDb system Click here for file
