# Supplementary material for: XenDB: Full length cDNA prediction and cross species mapping in Xenopus laevis
Source: BMC Genomics. 2005 Sep 14;6:123. doi: 10.1186/1471-2164-6-123 (PMC1261260; doi:10.1186/1471-2164-6-123)
Supplement: Additional File 2 — Table S1, Distribution of EST sequences in the analysis based on the annotated tissue source for the preparation of the library. (NOTE: annotations are imported directly from GenBank entries and are dependent on the original annotation.) [file 1471-2164-6-123-S2.doc]

Table S1: Most abundant tissue types in the X. *laevis* data set as annotated in GenBank entries

| Tissue Type | **No of Sequences** |
| --- | --- |
| N/A | 120653 |
| whole embryo | 105295 |
| Egg, oocyte libraries | 59533 |
| Gastrula libraries | 22490 |
| Neurula Libraries | 8164 |
| Embryos, stages 19-26 | 6658 |
| egg, subtracted by stage 13-17 animal cap | 3806 |
| embryo, animal cap | 2907 |
| head, stage 30 | 2761 |
| pooled embryos (stage 10-14) | 2672 |
| dorsal lip | 2491 |
| Normal testis | 2407 |
| total brain tissue | 1917 |
| whole tadpole | 1855 |
| Embryo, stage 31/32, *Xenopus* | 1418 |
| anterior endomesoderm | 1044 |
| egg, subtracted by stage 19-26 animal cap | 489 |
| Cornea | 233 |
