# Supplementary material for: XenDB: Full length cDNA prediction and cross species mapping in Xenopus laevis
Source: BMC Genomics. 2005 Sep 14;6:123. doi: 10.1186/1471-2164-6-123 (PMC1261260; doi:10.1186/1471-2164-6-123)
Supplement: Additional File 3 — Table S2: The 20 most abundant developmental stage annotations in the X. laevis data set as annotated in GenBank: Distribution of EST sequences in the analysis based on the annotated developmental stage of the source library. (NOTE: annotations are imported directly from GenBank entries and are dependent on the original annotation.) [file 1471-2164-6-123-S3.doc]

**Table S2: The 20 most abundant developmental stage annotations in the X. *laevis* data set as annotated in GenBank**

| Developmental Stage | No of Sequences |
| --- | --- |
| N/A | 127219 |
| Adult | 43436 |
| Gastrula | 42394 |
| Neurula | 30094 |
| Embryo, Stage 19-25 | 40346 |
| Embryo, Stage 31-32 | 20745 |
| Metamorphosis Stage 62 | 13894 |
| Metamorphosis 50-53 | 10648 |
| Tadpole | 1950 |
| Embryo | 60 |
