# Supplementary material for: XenDB: Full length cDNA prediction and cross species mapping in Xenopus laevis
Source: BMC Genomics. 2005 Sep 14;6:123. doi: 10.1186/1471-2164-6-123 (PMC1261260; doi:10.1186/1471-2164-6-123)
Supplement: Additional File 4 — Table S3: The 30 most abundant Clone Libraries in the X. laevis data set as determined by the GenBank annotation. (NOTE: annotations are imported directly from GenBank entries and are dependent on the original annotation.) [file 1471-2164-6-123-S4.doc]

Table S3: The 30 most abundant Clone Libraries in the X. *laevis* data set as determined by the GenBank annotation

|  | |
| --- | --- |
| Clone Library | **No of sequences** |
| NIBB Mochii normalized *Xenopus* early gastrula library | 40476 |
| NIBB Mochii normalized *Xenopus* tailbud library | 35548 |
| NIBB Mochii normalized *Xenopus* neurula library | 28720 |
| NICHD_XGC_Emb4 | 22163 |
| Blackshear/Soares normalized *Xenopus* egg library | 19023 |
| NICHD_XGC_Sp1 | 14997 |
| NICHD_XGC_Emb1 | 14683 |
| NICHD_XGC_Tad2 | 14041 |
| NICHD_XGC_OO1 | 13948 |
| NICHD_XGC_Eye1 | 11981 |
| NICHD_XGC_Brn1 | 10762 |
| NICHD_XGC_Ov1 | 10692 |
| NICHD_XGC_Tad1 | 10629 |
| Wellcome CRC pSK egg | 9491 |
| NICHD_XGC_Kid1 | 9458 |
| NICHD_XGC_Lu1 | 5641 |
| NICHD_XGC_Emb2 | 5425 |
| NICHD_XGC_He1 | 4293 |
| Wellcome CRC pRN3 St13 17 egg animal cap | 3806 |
| *Xenopus* *laevis* gastrula non normalized | 3659 |
| NICHD_XGC_Li1 | 3620 |
| *Xenopus* *laevis* oocyte | 3598 |
| *Xenopus* *laevis* unfertilized egg cDNA library | 2941 |
| NICHD_XGC_Emb3 | 2927 |
| Wellcome CRC pSK animal cap | 2907 |
| Harland stage 19-23 | 2881 |
| N/A | 2859 |
| RIKEN *Xenopus* egg | 2836 |
| Wellcome CRC pRN3 head | 2761 |
| Kirschner embryo St10 14 | 2672 |
| Wellcome CRC pcDNAI egg | 2631 |
| *Xenopus* *laevis* oocyte non normalized | 2583 |
| Wellcome CRC pRN3 dorsal lip | 2487 |
| Wellcome CRC pRN3 St19 26 | 2456 |
| Wellcome CRC pRN3 oocyte | 2446 |
| NICHD_XGC_Te1 | 2407 |
| *Xenopus* EST library | 1917 |
| *Xenopus* *laevis* tadpole stage 24 | 1852 |
| normalized *Xenopus* *laevis* gastrula | 1609 |
| Wellcome CRC pSK St 10 5 | 1493 |
