# Supplementary figures and images for: XenDB: Full length cDNA prediction and cross species mapping in Xenopus laevis
Source: BMC Genomics. 2005 Sep 14;6:123. doi: 10.1186/1471-2164-6-123 (PMC1261260; doi:10.1186/1471-2164-6-123)

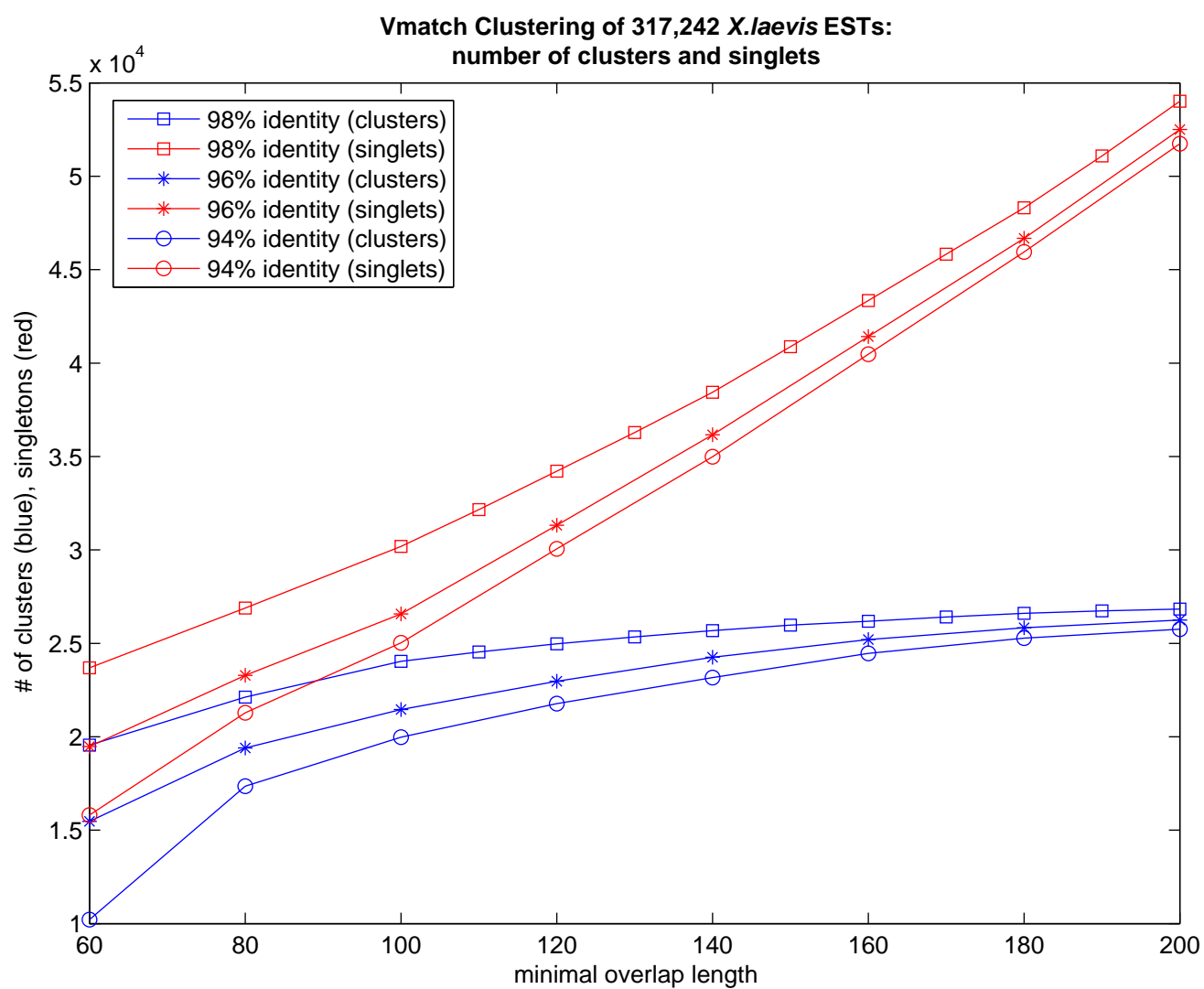

Supplement: Additional File 1 — Figure S1, Effect of Parameter Variation on EST Clustering: Masked and trimmed EST sequences were clustered using the Vmatch algorithm using different overlap length and percentage identity values. The total number of clusters (blue) and the number of singletons (red) are plotted against the minimal overlap length. Values were plotted at different percentage identities (squares 98%, stars 96%, circles 94%). [file 1471-2164-6-123-S1.pdf]
