# Supplementary material for: XenDB: Full length cDNA prediction and cross species mapping in Xenopus laevis
Source: BMC Genomics. 2005 Sep 14;6:123. doi: 10.1186/1471-2164-6-123 (PMC1261260; doi:10.1186/1471-2164-6-123)
Supplement: Additional File 5 — Table S4: Sizes of protein sets used for sequence analysis of clustered sequences. [file 1471-2164-6-123-S5.doc]

Table S4: Sizes of protein sets used for sequence analysis of clustered sequences

| **Organism** | **Source** | **Number of sequences** |
| --- | --- | --- |
| *H. sapiens* | IPI | 41,809 |
| *M. musculus* | IPI | 40,865 |
| *R. norvegicus* | IPI | 33,034 |
| *C. elegans* | UniProt | 22,866 |
| *D. melanogaster* | UniProt | 16,159 |
| X. laevis | Genbank (NR) | 7,917 |
| X. tropicalis | Genbank (NR) | 694 |
| GenBank(NR) | NCBI | 1,578,346 |
