# Supplementary material for: XenDB: Full length cDNA prediction and cross species mapping in Xenopus laevis
Source: BMC Genomics. 2005 Sep 14;6:123. doi: 10.1186/1471-2164-6-123 (PMC1261260; doi:10.1186/1471-2164-6-123)
Supplement: Additional File 6 — file containing the SAGE database query used in the glioblastoma and astrocytoma analysis. [file 1471-2164-6-123-S6.htm]

 


SAGE DGED Results


|  |
| --- |
|  |

|  |  |  |
| --- | --- | --- |
| **CGAP HOW TO** | | |


|  |  |  |  |  |  |  |  |  |  |  |  |  |  |  |  |  |  |  |  |  |  |  |  |  |  |  |  |  |  |  |  |  |  |  |  |  |  |  |  |  |  |  |  |  |  |  |  |  |  |  |  |  |  |  |  |  |  |  |  |  |  |  |  |  |  |  |  |  |  |  |  |  |  |  |  |  |  |  |  |  |  |  |  |  |  |  |  |  |  |  |  |  |  |  |  |  |  |  |  |  |  |  |  |  |  |  |  |  |  |  |  |  |  |  |  |  |  |  |  |  |  |  |  |  |  |  |  |  |  |  |  |  |  |  |  |  |  |  |  |  |  |  |  |  |  |  |  |  |  |  |  |  |  |  |  |  |  |  |  |  |  |  |  |  |  |  |  |  |  |  |  |  |  |  |  |  |  |  |  |  |  |  |  |  |  |  |  |  |  |  |  |  |  |  |  |  |  |  |  |  |  |  |  |  |  |  |  |  |  |  |  |  |  |  |  |  |  |  |  |  |  |  |  |  |  |  |  |  |  |  |  |  |  |  |  |  |  |  |  |  |  |  |  |  |  |  |  |  |  |  |  |  |  |  |  |  |  |  |  |  |  |  |  |  |  |  |  |  |  |  |  |  |  |  |  |  |  |  |  |  |  |  |  |  |  |  |  |  |  |  |  |  |  |  |  |  |  |  |  |  |  |  |  |  |
| --- | --- | --- | --- | --- | --- | --- | --- | --- | --- | --- | --- | --- | --- | --- | --- | --- | --- | --- | --- | --- | --- | --- | --- | --- | --- | --- | --- | --- | --- | --- | --- | --- | --- | --- | --- | --- | --- | --- | --- | --- | --- | --- | --- | --- | --- | --- | --- | --- | --- | --- | --- | --- | --- | --- | --- | --- | --- | --- | --- | --- | --- | --- | --- | --- | --- | --- | --- | --- | --- | --- | --- | --- | --- | --- | --- | --- | --- | --- | --- | --- | --- | --- | --- | --- | --- | --- | --- | --- | --- | --- | --- | --- | --- | --- | --- | --- | --- | --- | --- | --- | --- | --- | --- | --- | --- | --- | --- | --- | --- | --- | --- | --- | --- | --- | --- | --- | --- | --- | --- | --- | --- | --- | --- | --- | --- | --- | --- | --- | --- | --- | --- | --- | --- | --- | --- | --- | --- | --- | --- | --- | --- | --- | --- | --- | --- | --- | --- | --- | --- | --- | --- | --- | --- | --- | --- | --- | --- | --- | --- | --- | --- | --- | --- | --- | --- | --- | --- | --- | --- | --- | --- | --- | --- | --- | --- | --- | --- | --- | --- | --- | --- | --- | --- | --- | --- | --- | --- | --- | --- | --- | --- | --- | --- | --- | --- | --- | --- | --- | --- | --- | --- | --- | --- | --- | --- | --- | --- | --- | --- | --- | --- | --- | --- | --- | --- | --- | --- | --- | --- | --- | --- | --- | --- | --- | --- | --- | --- | --- | --- | --- | --- | --- | --- | --- | --- | --- | --- | --- | --- | --- | --- | --- | --- | --- | --- | --- | --- | --- | --- | --- | --- | --- | --- | --- | --- | --- | --- | --- | --- | --- | --- | --- | --- | --- | --- | --- | --- | --- | --- | --- | --- | --- | --- | --- | --- | --- | --- | --- | --- | --- | --- | --- | --- | --- | --- | --- | --- | --- | --- | --- | --- | --- | --- | --- | --- | --- | --- | --- | --- | --- | --- | --- | --- | --- |
| **SAGE Genie Tools**  - Anatomic Viewer- DGED- Absolute Level Lister- Library Finder- Downloads   **Related Links**  - SAGEmap xProfiler - SAGEmap vNorthern - SAGE (JHU)   **Quick Links:**  - ICG- NCI Home- NCICB Home- NCBI Home- OCG | Pool A and B Setup for DGED                              1. Select statistical parameters: |  |  |  |    | --- | --- | --- |    | F (Expression factor) |  | Range of F: any number greater than or equal to 1. Results are reported only when odds ratio is significantly greater than F or significantly less than 1/F. |    | P (Significance filter) |  | Range of P: 0 (show only most significant results) to 1 (show all results). Significance of results depends on value of F. |      - Enter a chromosome (1, 2, 3, ..., 21, 22, X, Y) on which you want to display the only tags in genes.              Chromosome            - Review Pool A and B libraries and remove unacceptable libraries        by clearing their checked boxes.          - To refresh the page with the modified settings,          press "Update Defaults", which will allow you to          use the "Save As" feature on your browser.          See Saving a DGED Setup.            - To obtain results, press "Submit Query".              - To revert to the original settings, press "Reset Selection".            |  |  |  |  |  | | --- | --- | --- | --- | --- | | **Pool** | | **Library Name** | **Tags** | **Keywords** | | **A** | **B** | |  |  | SAGE\_Brain\_astrocytoma\_grade\_III\_B\_R140 | 118733 | brain, astrocytoma grade III, bulk, short SAGE, male, adult | |  |  | SAGE\_Brain\_ependymoma\_B\_1394 | 56314 | brain, ependymoma, short SAGE, non-normalized, bulk | |  |  | SAGE\_Brain\_glioblastoma\_B\_pooled | 56428 | brain, glioblastoma multiforme, non-normalized, short SAGE, bulk | |  |  | SAGE\_Brain\_medulloblastoma\_B\_98-09-P558 | 85984 | brain, medulloblastoma, bulk, short SAGE, female | |  |  | SAGE\_Brain\_medulloblastoma\_B\_H484 | 57469 | brain, medulloblastoma, bulk, short SAGE, male | |  |  | SAGE\_Brain\_glioblastoma\_B\_R336 | 102322 | brain, glioblastoma multiforme, bulk, short SAGE, adult, male | |  |  | SAGE\_Brain\_medulloblastoma\_B\_H1413 | 61853 | brain, medulloblastoma, bulk, short SAGE, male | |  |  | SAGE\_Brain\_medulloblastoma\_B\_H306 | 60454 | brain, medulloblastoma, bulk, short SAGE, female | |  |  | SAGE\_Brain\_astrocytoma\_grade\_II\_B\_H359 | 105764 | brain, non-normalized, bulk, astrocytoma grade II, short SAGE | |  |  | SAGE\_Brain\_astrocytoma\_grade\_III\_B\_R927 | 107344 | brain, astrocytoma grade III, bulk, short SAGE, adult, female | |  |  | SAGE\_Brain\_medulloblastoma\_B\_98-13-P301 | 45342 | brain, medulloblastoma, bulk, metastasis, short SAGE, male | |  |  | SAGE\_Brain\_ependymoma\_B\_R1023 | 122690 | brain, non-normalized, bulk, ependymoma, short SAGE | |  |  | SAGE\_Brain\_glioblastoma\_B\_R70 | 99099 | brain, glioblastoma multiforme, bulk, short SAGE, adult, female | |  |  | SAGE\_Brain\_astrocytoma\_grade\_III\_B\_H970 | 106982 | brain, astrocytoma grade III, bulk, adult, male, short SAGE | |  |  | SAGE\_Brain\_medulloblastoma\_B\_DL7 | 68392 | brain, medulloblastoma, bulk, short SAGE, male | |  |  | SAGE\_Brain\_fetal\_normal\_B\_S1 | 306283 | brain, normal, bulk, fetus, extracted short SAGE | |  |  | SAGE\_Brain\_normal\_substantia\_nigra\_B\_1 | 42498 | brain, substantia nigra, normal, bulk, extracted short SAGE | |  |  | SAGE\_Brain\_astrocytoma\_grade\_II\_B\_H530 | 102439 | brain, astrocytoma grade II, bulk, short SAGE, adult, female | |  |  | SAGE\_Brain\_astrocytoma\_grade\_II\_B\_H388 | 106285 | brain, non-normalized, bulk, astrocytoma grade II, short SAGE | |  |  | SAGE\_Brain\_medulloblastoma\_B\_C609 | 74612 | brain, medulloblastoma, bulk, short SAGE, male | |  |  | SAGE\_Brain\_ependymoma\_B\_R628 | 120431 | brain, non-normalized, bulk, ependymoma, short SAGE | |  |  | SAGE\_Brain\_ependymoma\_B\_R353 | 73822 | brain, non-normalized, bulk, ependymoma, short SAGE | |  |  | SAGE\_Brain\_normal\_cortex\_B\_pool6 | 62451 | cortex, normal, non-normalized, short SAGE, bulk | |  |  | SAGE\_Brain\_medulloblastoma\_B\_H876 | 67404 | brain, medulloblastoma, bulk, short SAGE, male | |  |  | SAGE\_Brain\_ependymoma\_B\_239 | 46653 | brain, ependymoma, short SAGE, non-normalized, bulk | |  |  | SAGE\_Brain\_ependymoma\_B\_R582 | 52189 | brain, non-normalized, bulk, ependymoma, short SAGE | |  |  | SAGE\_Brain\_oligodendroglioma\_B\_1001 | 32442 | cortex, oligodendroglioma, well differentiated, non-normalized, short SAGE, bulk | |  |  | SAGE\_Brain\_medulloblastoma\_B\_H1322 | 59498 | brain, medulloblastoma, metastasis, bulk, short SAGE, male | |  |  | SAGE\_Brain\_normal\_thalamus\_B\_1 | 24015 | normal, bulk, thalamus, non-normalized, short SAGE | |  |  | SAGE\_Brain\_medulloblastoma\_B\_H972 | 85376 | brain, medulloblastoma, bulk, short SAGE, male | |  |  | SAGE\_Brain\_ependymoma\_B\_H580 | 68614 | brain, bulk, ependymoma, adult, male, short SAGE | |  |  | SAGE\_Brain\_medulloblastoma\_B\_98-05-P040 | 89258 | brain, medulloblastoma, metastasis, bulk, short SAGE, male | |  |  | SAGE\_Brain\_medulloblastoma\_B\_H275 | 72318 | brain, medulloblastoma, bulk, short SAGE, adult, female | |  |  | SAGE\_Brain\_medulloblastoma\_B\_DL5 | 83671 | brain, medulloblastoma, bulk, short SAGE, male | |  |  | SAGE\_Brain\_normal\_peds\_cortex\_B\_H1571 | 77554 | brain, cortex, normal, non-normalized, short SAGE, bulk | |  |  | SAGE\_Brain\_ependymoma\_B\_R512 | 75379 | brain, ependymoma, bulk, short SAGE, female | |  |  | SAGE\_Brain\_ependymoma\_B\_R455 | 51825 | brain, non-normalized, bulk, ependymoma, short SAGE | |  |  | SAGE\_Brain\_astrocytoma\_grade\_I\_B\_H1043 | 75922 | brain, pilocytic, astrocytoma, bulk, non-normalized, short SAGE, astrocytoma, grade I | |  |  | SAGE\_Brain\_ependymoblastoma\_B\_819 | 33975 | brain, ependymoblastoma, short SAGE, non-normalized, bulk | |  |  | SAGE\_Brain\_astrocytoma\_grade\_II\_B\_H563 | 88568 | brain, non-normalized, bulk, astrocytoma grade II, short SAGE | |  |  | SAGE\_Brain\_ependymoma\_B\_R510 | 84073 | brain, ependymoma, bulk, short SAGE, female | |  |  | SAGE\_Brain\_medulloblastoma\_B\_97-05-P312 | 74295 | brain, medulloblastoma, bulk, short SAGE, male | |  |  | SAGE\_Brain\_ependymoma\_B\_1150 | 62373 | brain, ependymoma, short SAGE, non-normalized, bulk | |  |  | SAGE\_Brain\_normal\_cortex\_B\_BB542 | 94233 | cortex, normal, non-normalized, short SAGE, bulk | |  |  | SAGE\_Brain\_glioblastoma\_B\_H833 | 100600 | brain, glioblastoma multiforme, bulk, short SAGE, adult, male | |  |  | SAGE\_Brain\_glioblastoma\_B\_H1110 | 68986 | brain, glioblastoma multiforme, non-normalized, short SAGE, bulk | |  |  | SAGE\_Brain\_medulloblastoma\_B\_98-04-P117 | 32570 | brain, medulloblastoma, bulk, short SAGE, male | |  |  | SAGE\_Brain\_glioblastoma\_B\_GBM1062 | 59762 | brain, glioblastoma multiforme, bulk, non-normalized, short SAGE | |  |  | SAGE\_Brain\_oligodendroglioma\_B\_H988 | 27864 | cortex, oligodendroglioma, well differentiated, non-normalized, short SAGE, bulk | |  |  | SAGE\_Brain\_astrocytoma\_grade\_III\_B\_H1020 | 51573 | brain, anaplastic, astrocytoma, bulk, non-normalized, short SAGE, astrocytoma, grade III | |  |  | SAGE\_Brain\_medulloblastoma\_B\_98-05-P608 | 48451 | medulloblastoma, cerebellum, bulk, non-normalized, short SAGE | |  |  | SAGE\_Brain\_medulloblastoma\_B\_96-04-P019 | 52645 | medulloblastoma, cerebellum, bulk, non-normalized, short SAGE | |  |  | SAGE\_Brain\_normal\_cerebellum\_B\_1 | 50385 | normal, cerebellum, bulk, non-normalized, short SAGE | |  |  | SAGE\_Brain\_medulloblastoma\_B\_98-04-P494 | 43068 | medulloblastoma, cerebellum, bulk, non-normalized, short SAGE | |  |  | SAGE\_Brain\_medulloblastoma\_B\_1273 | 38614 | medulloblastoma, cerebellum, bulk, non-normalized, short SAGE | |  |  | SAGE\_Brain\_medulloblastoma\_B\_97-05-P015 | 69971 | medulloblastoma, cerebellum, bulk, non-normalized, short SAGE | |  |  | SAGE\_Brain\_normal\_cerebellum\_B\_BB542 | 40500 | normal, cerebellum, bulk, non-normalized, short SAGE | |  |  | SAGE\_Spinal\_cord\_normal\_B\_1 | 54785 | spinal cord, bulk, normal, non-normalized, short SAGE | |

---

If you have any questions, comments, or need information about CGAP,
please contact the 
NCI CGAP Help Desk.
